# Horizontal transfer and phylogenetic distribution of the immune evasion factor *tarP*

**DOI:** 10.3389/fmicb.2022.951333

**Published:** 2022-10-28

**Authors:** David Gerlach, Raphael N. Sieber, Jesper Larsen, Janes Krusche, Cristina De Castro, Juliane Baumann, Antonio Molinaro, Andreas Peschel

**Affiliations:** ^1^Interfaculty Institute of Microbiology and Infection Medicine, Infection Biology Section, University of Tübingen, Tübingen, Germany; ^2^German Center for Infection Research (DZIF), Partner Site Tübingen, Tübingen, Germany; ^3^Cluster of Excellence EXC2124 Controlling Microbes to Fight Infection, University of Tübingen, Tübingen, Germany; ^4^Statens Serum Institut, Copenhagen, Denmark; ^5^Department of Chemical Sciences, University of Naples, Naples, Italy

**Keywords:** *Staphylococcus aureus*, bacteriophage, teichoic acids, bacterial genomics, horizontal gene transfer

## Abstract

Methicillin-resistant *Staphylococcus aureus* (MRSA), a major human pathogen, uses the prophage-encoded *tarP* gene as an important immune evasion factor. TarP glycosylates wall teichoic acid (WTA) polymers, major *S. aureus* surface antigens, to impair WTA immunogenicity and impede host defence. However, *tarP* phages appear to be restricted to only a few MRSA clonal lineages, including clonal complexes (CC) 5 and 398, for unknown reasons. We demonstrate here that *tarP*-encoding prophages can be mobilized to lysogenize other *S. aureus* strains. However, transfer is largely restricted to closely related clones. Most of the non-transducible clones encode *tarM*, which generates a WTA glycosylation pattern distinct from that mediated by TarP. However, *tarM* does not interfere with infection by *tarP* phages. Clonal complex-specific Type I restriction-modification systems were the major reasons for resistance to *tarP* phage infection. Nevertheless, *tarP* phages were found also in unrelated *S. aureus* clones indicating that *tarP* has the potential to spread to distant clonal lineages and contribute to the evolution of new MRSA clones.

## Introduction

In order to maintain and optimize its role as a major human pathogen, *Staphylococcus aureus* relies on horizontal gene transfer (HGT), by which virulence factors and resistance genes are exchanged to create new clonal lineages with increased virulence, resistance, or dissemination capacities (Lindsay, 2010) or with new host specificities (Oliveira et al., 2014; Sheppard et al., 2018). Bacteriophages, bacterial viruses, are the crucial vehicles of HGT in *S. aureus* (Xia and Wolz, 2014). Siphophages often form prophages, which can integrate into host bacterial chromosomes to contribute up to 20% of the bacterial genomic content. Some of these phages contain genes and gene clusters that interfere with host immune function, including the immune evasion cluster 1 (IEC-1, from now on referred to as “IEC”) that enables *S*. *aureus* to evade the human innate immune response (Richardson et al., 2018) and the *tarP* gene, which impairs the immunogenicity of wall teichoic acid (WTA), a major *S. aureus* surface antigen (Gerlach et al., 2018). Moreover, phages can mobilize resistance determinants such as the *mecA* gene of methicillin-resistant *S. aureus* (MRSA) (Maslanova et al., 2013; Scharn et al., 2013).

Phages use the species and clone-specific structure of WTA to bind to and infect appropriate bacterial host cells (Winstel et al., 2013). In most *S. aureus,* WTA is a surface glycopolymer consisting of polyribitol phosphate (poly-RboP) repeats that are linked covalently to the peptidoglycan *via* a linkage unit (Weidenmaier and Peschel, 2008; Brown et al., 2013). RboP-WTA is modified with N-acetylglucosamine (GlcNAc) residues, which can be linked in different conformations, thereby governing the susceptibility of *S. aureus* to specific phage groups and the immunogenicity of WTA (Winstel et al., 2014; Gerlach et al., 2018; Ingmer et al., 2019). Most *S. aureus* isolates encode the housekeeping GlcNAc transferase (GT) *tarS,* which confers a β-1,4-GlcNAc-RboP glycosylation pattern (Brown et al., 2012). Some *S. aureus* isolates express another genome-encoded GT, TarM, which attaches α-1,4-GlcNAc residues to RboP (Xia et al., 2010). Like *tarS*, *tarM* does not appear to be associated with either phages or other mobile genetic elements (MGEs) (Li et al., 2015). In contrast, the phage-encoded GT TarP uses the same substrate as TarS and TarM (UDP-GlcNAc) but attaches GlcNAc to RboP with a β-1,3 configuration (Gerlach et al., 2018). We previously demonstrated that *tarP* is prevalent in some of the major hospital-associated (HA), MRSA sequence types (STs), 5 and 225, both of which belong to clonal complex (CC) 5 and the major livestock-associated (LA) lineage CC398, contributing to the immune evasion capacities of these clones (Gerlach et al., 2018). It has remained unclear though why *tarP* is absent from many other MRSA clones and whether it could potentially spread to give rise to new pathogen lineages.

Horizontal transfer of phages and other MGEs is not only restricted by WTA modification, but also by Type I restriction-modification (RM) (Oliveira et al., 2014, 2016). The importance of Type I RM systems is reflected by their high abundance in almost all *S. aureus* genomes along with clone-specific conservation but high inter-clonal diversity of specific RM types (Sadykov, 2016). Three different host specificity determinants make up Type I RM systems: the nuclease HsdR, designated as SauI in *S. aureus* (Waldron and Lindsay, 2006); the DNA methyltransferase HsdM; and HsdS that confers specificity *via* two DNA target recognition domains to HsdR, and HsdM in a clonal complex-specific manner (Wilson and Murray, 1991).

Here, we comprehensively analyzed all genomes of *S. aureus* deposited to public databases for the presence of *tarP* and its association with *tarS and tarM.* We investigated genetic diversity of *tarP* phages and their transmission dynamics between different hosts and demonstrate that its horizontal transfer is not affected by *tarM* or *tarS*-mediated WTA modifications but by incompatibility between Type I RM systems of bacterial phage donor and acceptor clones.

## Materials and methods

### Bacterial strains and growth conditions

Bacterial strains used for this study can be found in Supplementary Table S3. *S. aureus* was cultivated in tryptic soy broth (TSB) and *Escherichia coli* in Lysogeny broth (LB), in the presence of appropriate antibiotics (chloramphenicol 10 μg/ml, ampicillin 100 μg/ml) at 37°C. For the calculation of growth rates in TSB or RPMI media bacterial densities were monitored at 595 nm using an Epoch 2 (BioTek) device. The maximum slope was extracted as growth rate from the logarithmic growth curve after background correction.

### Molecular biology

All oligonucleotide primers used for this study are listed in Supplementary Table S4. Modification of *tarP* phage genomes with antibiotic resistance genes or marker-less deletion of *tarP* and *tarS* were established by using the *E. coli*/*S. aureus* shuttle vector pBASE6 plasmid and procedures described previously (Geiger et al., 2012).

*tarP* was deleted in NCTC13132 using the previously published plasmid pIMAY_*tarP* (Gerlach et al., 2018). Mutagenesis was accomplished as described recently (Monk et al., 2015).

*hsdR* (SAUSA300_0196) was amplified from genomic DNA of either USA300 (intact *hsdR*) or RN4220 (non-functional *hsdR*) with primers containing restriction sites BamHI and SacI and ligated into shuttle vector pRB474. The resulting plasmids were subsequently used to transform the restriction-deficient strain RN4220 by electroporation.

### Phage-biological methods

#### Induction of prophages

Bacterial strains harboring prophages were grown in TSB to an OD 595 nm of 0.4–0.5 and prophages were induced by addition of 1 μg/ml mitomycin C for 4 h at 30°C with slow agitation and eventually overnight incubation at ambient temperature without shaking. Phages were purified by removing bacteria and cell debris by centrifugation and filtration with a pore size of 0.22 μm. Phage titers of the obtained lysates were determined by adding appropriate dilutions in soft ager to agar plates with susceptible test strains and counting of phage plaques. For phage ΦN315, indicator strain *S. aureus* R5 was used, for phage ΦSa1int-*tarP* and ΦSebago_int-*tarP S. aureus* RN4220.

#### Phage transfer

Lysogenization by induced phages was analyzed by diluting overnight cultures of bacterial recipient strains to an OD_595 nm_ of 0.1 and mixing 200 μl of bacterial suspension with 100 μl phage lysate followed by incubation for 15 min at 37°C with mild agitation. As an exception, for transfer assays with restored *hsdR* in RN4220 (depicted in Figure 5), 100 μl bacteria were mixed with 200 μl phage suspension. Phage lysates were then plated on TSB plates containing the appropriate antibiotic (either 3 μg/ml tetracycline for ΦN315 or 2.5 μg/ml erythromycin for ΦSa1int-*tarP* and ΦSebago_int-*tarP*) and incubated for 2 days at 37°C. Successful lysogenization by *tarP* phages was confirmed by molecular typing of the *tarP* gene or, in case of ΦN315, additionally by altered hemolysis phenotype on TSB blood plates, because this phage integrates into the beta-hemolysin gene.

#### Phage susceptibility assay

Susceptibility of bacterial strains to phages was determined by the previously described soft agar overlay method (Xia et al., 2010). Briefly, bacterial overnight cultures were diluted to an OD_595 nm_ of 0.1 and 200 μl were mixed with 5 ml prewarmed soft ager (1% tryptone, 0.5% yeast extract, 0.5% NaCl, 0.1% K_2_HPO_4_, 0.1% glucose, 0.4% agar) and poured in TSB plates. Phage suspensions (10 μl) were dropped onto the soft agar and incubated at 37°C overnight.

### WTA isolation

Isolation of WTA was conducted as previously described (Gerlach et al., 2018). Briefly, WTA was isolated from stationary-phase bacteria grown in BM broth supplemented with extra glucose (1% tryptone, 0.5% yeast extract, 0.5% NaCl, 0.1% K2HPO4, 0.25% glucose). After repeated washing of bacterial cells in AA buffer (20 mM, ammonium acetate pH 4.8) cells were disrupted in a cell mill (Euler biotechnologies), and cell lysates were treated with DNAse (40 U/ml) and RNAse (80 U/ml) at 37°C for at least 6 h. Subsequently cell lysates were supplement with 2% SDS, incubated at 65°C for 60 min, and ultrasonicated for 15 min. After removal of SDS by extensive washing of cell walls with AA buffer, WTA was released from the insoluble cell wall fraction by incubation with 5% trichloroacetic acid for 4 h at 60°C. The pH of the WTA solution was adjusted to pH 4–5 and WTA was dialyzed against distilled water using a Spectra/Por3 dialysis membrane (MWCO of 3.5 kDa; VWR International GmbH, Darmstadt).

### Analysis of WTA

Phosphorus and GlcNAc content of WTA was determined by the method of Chen and Warner (1956) and Smith (1979), respectively.

### NMR data collection

All NMR experiments were carried out as described previously (Speciale et al., 2022). Briefly, samples (about 7–10 mg) were solved in D_2_O (550 μl) and measured at 298 K with a Bruker DRX-600 spectrometer equipped with a cryo-probe by using standard Bruker software. Chemical shift of spectra was measured in ppm relative to internal acetone (2.225 and 31.45 ppm). The spectral width was set to 10 ppm and the frequency carrier placed at the residual HOD peak, suppressed by presaturation. Two-dimensional spectra (TOCSY with a mixing time of 100 ms, gHSQC, gHMBC, and HSQC-TOCSY with a mixing time of 100 ms) were measured for all samples. For NCTC13132 Δ*tarP* and RN4220 ΦN135, additional spectra were acquired. HSQC-TOCSY with a mixing time of 20 ms was recorded for both, while a T-ROESY (350 ms of mixing time) and a NOESY (200 ms of mixing time) spectra were acquired for RN4220 ΦN135 and NCTC13132 Δ*tarP*, respectively.

For all the experiments, 512 free induction decays (FIDs) of 2,048 complex data points were collected; for homonuclear spectra, 32 scans per FID were acquired; heteronuclear ^1^H-^13^C spectra were measured in the ^1^H-detected mode, gHSQC spectrum was acquired with 40 scans per FID, gHMBC and HSQC-TOCSY scans doubled, or tripled, those of the gHSQC spectrum, respectively. During processing, each data matrix was zero-filled in both dimensions to give a matrix 4 K × 2 K points and was resolution-enhanced in both dimensions by a cosine-bell function before Fourier transformation; data processing and analysis was performed with Bruker Topspin 3 program.

Additional information on the NMR analysis procedure can be found in the Supplementary material.

### Bioinformatic analysis

General analysis of the datasets occurred using R (Version 4.02) or GraphPad Prism.

#### Analysis of restriction modification systems

Restriction modification patterns were analyzed using a custom R script to survey MGEs for described recognition sequences of Type-I restriction modification systems (Cooper et al., 2017).

#### Genomic analysis of gene presence-absence

All available *S. aureus* genomes were downloaded from the RefSeq Genome Database (accessed 10. December 2020) (O’Leary et al., 2016) and sequence-typed using the MLST software^1^ based on information stored on the PubMLST website (Jolley and Maiden, 2010). BLASTN searches (Altschul et al., 1990) were performed using reference sequences *S. aureus* COL (Genbank accession no. NC_002951) for *mecA*, *tarS,* and *tarM*, *S. aureus* N315 (Genbank accession no. NC_002745) for *tarP*, *S. aureus* LGA251 (Genebank accession no. NC_017349) for *mecC,* and Newman (Genbank accession no. NC_009641) for *scn* (an indicator of the IEC element) and the Sa3int. The genes were considered present when showing at least 90% length and 90% identity to the reference gene, except for *tarP*, where 80% identity was considered sufficient in order to account for a rare *tarP* variant with 82% identity to the reference.

*tarP*-associated integrase genes were identified by extraction and annotation of the 2-kb flanking regions in both directions for all *tarP* genes. Annotation was performed in Geneious Prime 2019 (Biomatters Ltd.) from an in-house collection of integrase genes based on Goerke et al. (2009).

#### Phylogenetic analysis of selected phyla

The contigs of selected CC’s or ST’s were used to call single nucleotide polymorphisms (SNPs) using NASP (Sahl et al., 2016). To remove SNPs falling into regions of putative recombination, Gubbins (Croucher et al., 2015) was used using default parameters. The maximum likelihood phylogenies were then established using IQTREE (Nguyen et al., 2015) using a GTR model of substitution and annotated using package ggtree (Yu et al., 2016) in R version 3.6. The respective phylogeny files are deposited as Supplementary material: Data sheet 1 – CC1; Data sheet 2 – CC45; Data sheet 3 – ST5.

## Results

### *tarP* is confined to distinct clonal lineages

**Figure 1:**
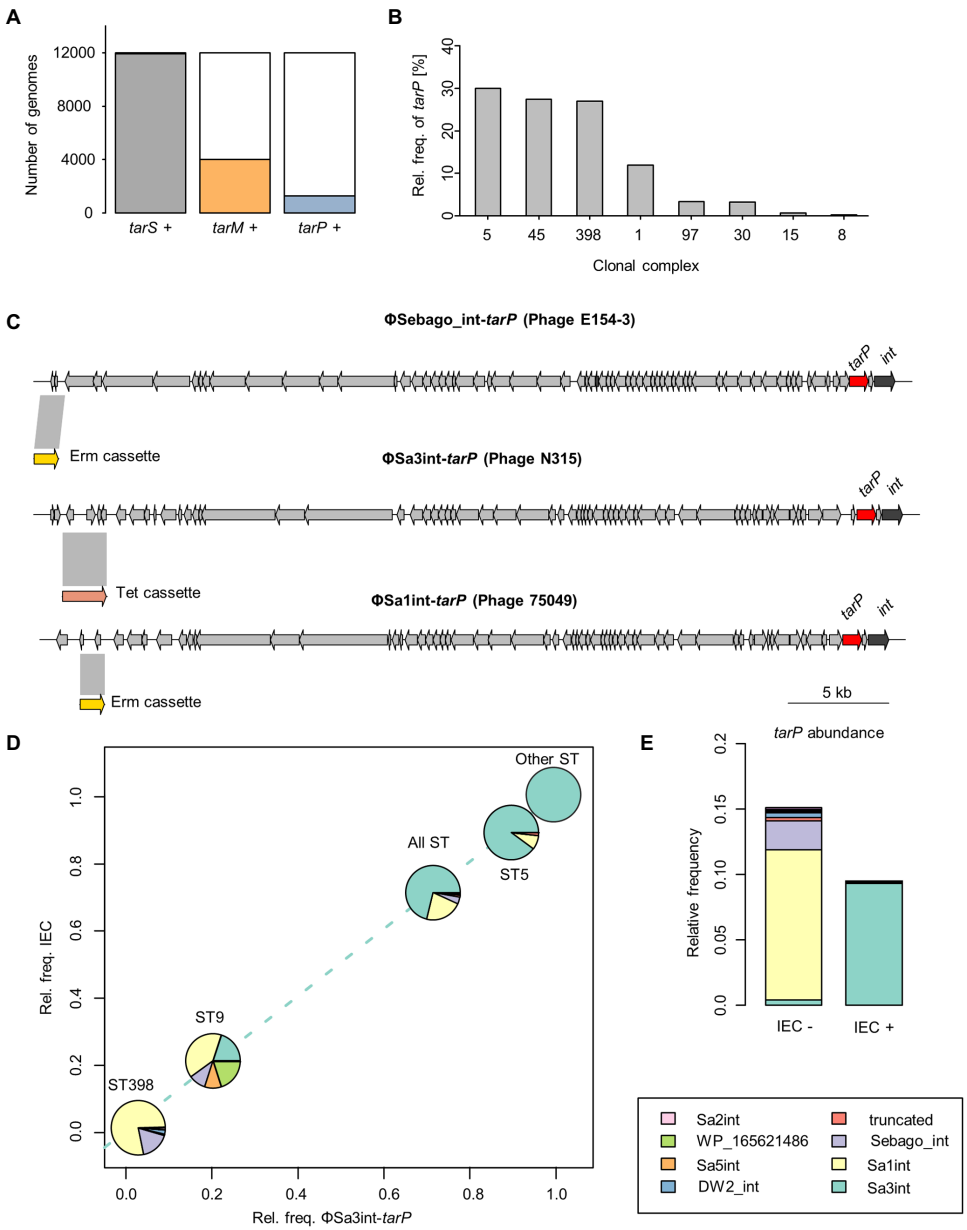
Distribution of *tarP* phages across *Staphylococcus aureus* isolates. **(A)** Absolute frequency of RboP WTA glycosyltransferases (GT). **(B)** Relative frequency in percent of *tarP* in clonal complexes. Clonal complexes were used to summarize ST. **(C)** Genetic structure of manipulated *tarP* phages, presented in this work, indicating location of *tarP*, integrase (red) and the respective, inserted antibiotic resistance marker (yellow/pink). **(D)** *tarP* phage distribution of main *tarP*-encoding ST (other STs comprise 45, 180, 188, 225, 228, and 3,410). Each ST is placed according to its relative frequencies of IEC and Sa3int-*tarP* phages. **(E)** Relative frequency of *tarP* in relation to IEC. *tarP*-associated phage integrases are indicated by the same colours as in panel **D**.

*tarS* was present in 99% of the 11,984 *S. aureus* genomes present in the NCBI Reference Sequence Database (Figure 1A). In agreement with our previous findings, *tarP* was most frequently present in CC5 and CC398 (30 and 27% of genomes, respectively; Table 1, Figure 1B). *tarP* was also prevalent in CC45 and CC1 (27 and 12% of the clones, respectively). In addition, *tarP* was found in CC8, CC15, CC30, and CC97, albeit at low frequencies (range 0.2–3.2%). Finally, *tarP* was present in few clones with very small numbers of available genomes (Table 1).

**Table 1:** Abundance of *tarP*-positive *S. aureus* sequence types (MLST) in the used BLAST dataset.

Clonal complex/MLST		Total number of genomes	Number of *tarP*-containing genomes	Percentage of *tarP-*containing genomes
**CC1/**	**All**	**268**	**32**	**11.9**
	9	176	10	5.7
	188	90	20	22.2
	1,207	2	2	100
**CC5/**	**All**	**2,718**	**815**	**30.0**
	5	2,519	691	27.4
	125	3	2	66.7
	149	9	1	11.1
	180	20	20	100
	225	39	31	79.5
	228	58	16	27.6
	248	6	4	66.7
	634	1	1	100
	764	9	1	11.1
	1,011	7	7	100
	1,184	1	1	100
	1,186	1	1	100
	1,457	1	1	100
	1866	3	1	33.3
	2060	1	1	100
	2,389	2	2	100
	2,625	2	2	100
	2,890	1	1	100
	2,905	1	1	100
	3,005	1	1	100
	3,312	1	1	100
	3,410	29	25	86.2
	3,481	1	1	100
	3,541	1	1	100
	3,571	1	1	100
**CC8/**	**All**	**2,107**	**4**	**0.2**
	8	2068	1	0
	247	37	1	2.7
	828	2	2	100
**CC15/**	**15**	**162**	**1**	**0.6**
**CC30/**	**All**	**251**	**8**	**3.2**
	30	250	7	2.8
	2,864	1	1	100
**CC45/**	**All**	**270**	**74**	**27.4**
	45	269	73	27.1
	2,249	1	1	100
**CC97**	**97**	**121**	**4**	**3.3**
**CC398**	**398**	**900**	**243**	**27**
Singleton/	3,709	7	7	100
	3,706	3	3	100
	49	3	2	66.7
	88	64	2	3.1
	1,391	1	1	100
	151	30	1	3.3
	3,092	1	1	100
	3,796	1	1	100
	4,068	1	1	100

In order to assess the risk for the future transfer of *tarP* phages to other important MRSA clones we sought to understand the underlying principles of the discrete phylogenetic distribution of *tarP* phages. *tarP* has a conserved position in diverse phage genomes, close to the gene for the phage integrase (Figure 1C), which directs the insertion position of the prophage in the bacterial genome. Integrase gene types can be utilized to classify staphylococcal siphophages into more than 12 different families (Goerke et al., 2009). We sought to understand the diversity of *tarP* phages in the analyzed genomes by mapping the *tarP*-associated integrase type. Sa3int was the most frequent integrase associated with *tarP*, although we observed strong differences across different STs (Figure 1D). ΦSa3int also harbors the IEC element that enables *S*. *aureus* to evade the human innate immune response and is highly prevalent in human *S*. *aureus* clones, whereas it is virtually absent from *S*. *aureus* clones from non-human animals (Richardson et al., 2018). Accordingly, IEC was significantly enriched in *S. aureus* genomes of human origin (*p* < 10^−15^, Fisher’s exact test) (Supplementary Figure 1). *tarP* was usually located on ΦSa3int phages in *S*. *aureus* clones harboring the IEC element, whereas it was present on many different phages in *S*. *aureus* clones lacking the IEC element, including ΦSa1int, ΦSa2int, ΦSa5int, ΦSa7int, and the newly described ΦSebago (Klotz et al., 2019) that was previously misidentified as Sa9int (Figure 1D) (Gerlach et al., 2018). In addition, we observed that *tarP* is 40% more frequent in strains lacking the IEC element than in those with IEC (*p* < 10^−13^ by Fisher’s exact test), suggesting that *tarP* is more prevalent in *S*. *aureus* clones from non-human animals (Figure 1E).

To assess the functional mobility of *tarP* phages, we constructed phylogenies of ST5, CC1, and CC45 (Supplementary material). In CC45 *tarP* was confined to a deep-branching clade, located on ΦSa3int, which reflects a history of vertical spread with no horizontal transfer of *tarP phages* to isolates belonging to other clades. In CC1, *tarP* was associated with different phages and showed a more scattered distribution, supporting the notion that it has been introduced into CC1 on multiple occasions. In ST5, *tarP* was located on ΦSa3int phages but showed a more scattered distribution than in CC45, suggesting a history of multiple introductions and/or losses and, therefore, potentially dynamic WTA glycosylation alterations.

**Figure 2:**
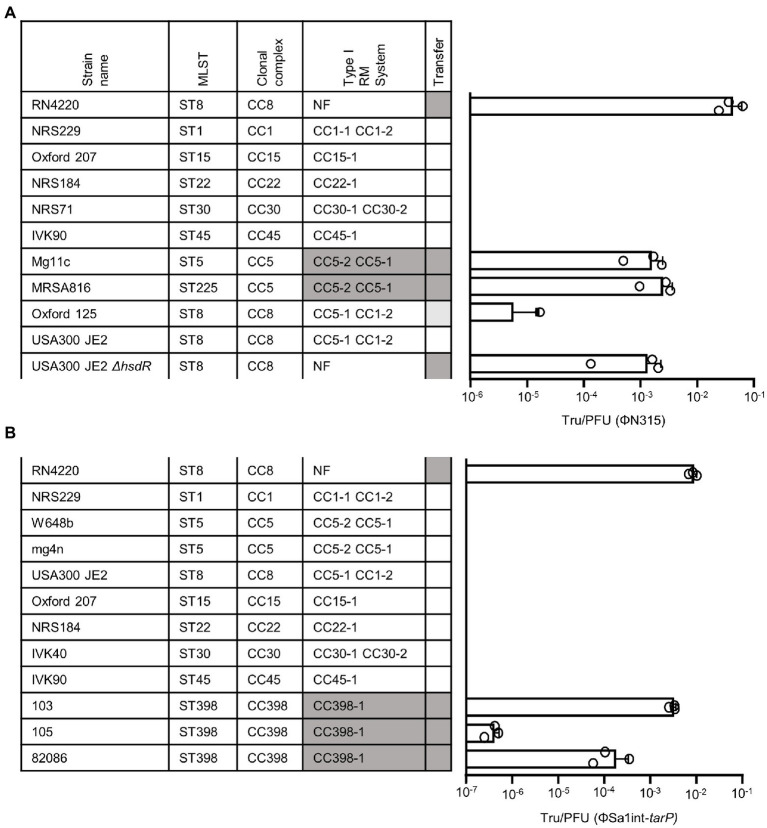
Transfer of *tarP* phages induced from different *S. aureus* backgrounds into different recipient strains. MLST, clonal complex, associated RM I system make-up, and successful transfer are indicated (NF, non-functional Type I RM system). RM systems that share identical make-up with the donor strain are marked by color. Transfer efficiency is depicted as Tru/PFU as mean + SD of 3 independent replicates. **(A)** Transfer of ΦN315 induced from a CC5 background. **(B)** Transfer of ΦSa1int-*tarP* induced from a CC398 background.

To study *tarP* phage dynamics in an experimental approach, we inserted antibiotic resistance markers into three dominant *tarP* prophages: ΦSa3int-*tarP* (ΦN315) in the genome of hospital-associated (HA)-MRSA N315 (CC5), ΦSa1int-*tarP* in LA-MRSA 75049 (ST398), and ΦSebago_int-*tarP* in LA-MRSA 70153 (ST398) (Figure 1C). The markers were placed by removing non-essential or non-coding genetic regions upstream of the phage holin/lysins genes to maintain phage activity. We attempted to mobilize the marked *tarP*-encoding phages ΦN315 and ΦSa1int-*tarP* by mitomycin C treatment and monitored lysogenization of the widely used phage-susceptible lab strain *S. aureus* RN4220. Both phage preparations could lysogenize RN4220 indicating that the two phages are indeed intact and can be mobilized (Figure 2). Next, we probed *S. aureus* isolates devoid of *tarP* prophages from different clonal complexes and backgrounds for susceptibility to the *tar* phages ΦSa3int*-tarP* (Figure 2A) and ΦSa1int-*tarP* (Figure 2B). In both cases we observed transfer only to acceptor strains that shared the same clonal background as the donor strains N315 (CC5) or 75,049 (CC398).

### *tarP* phages require glycosylated WTA for binding

**Figure 3:**
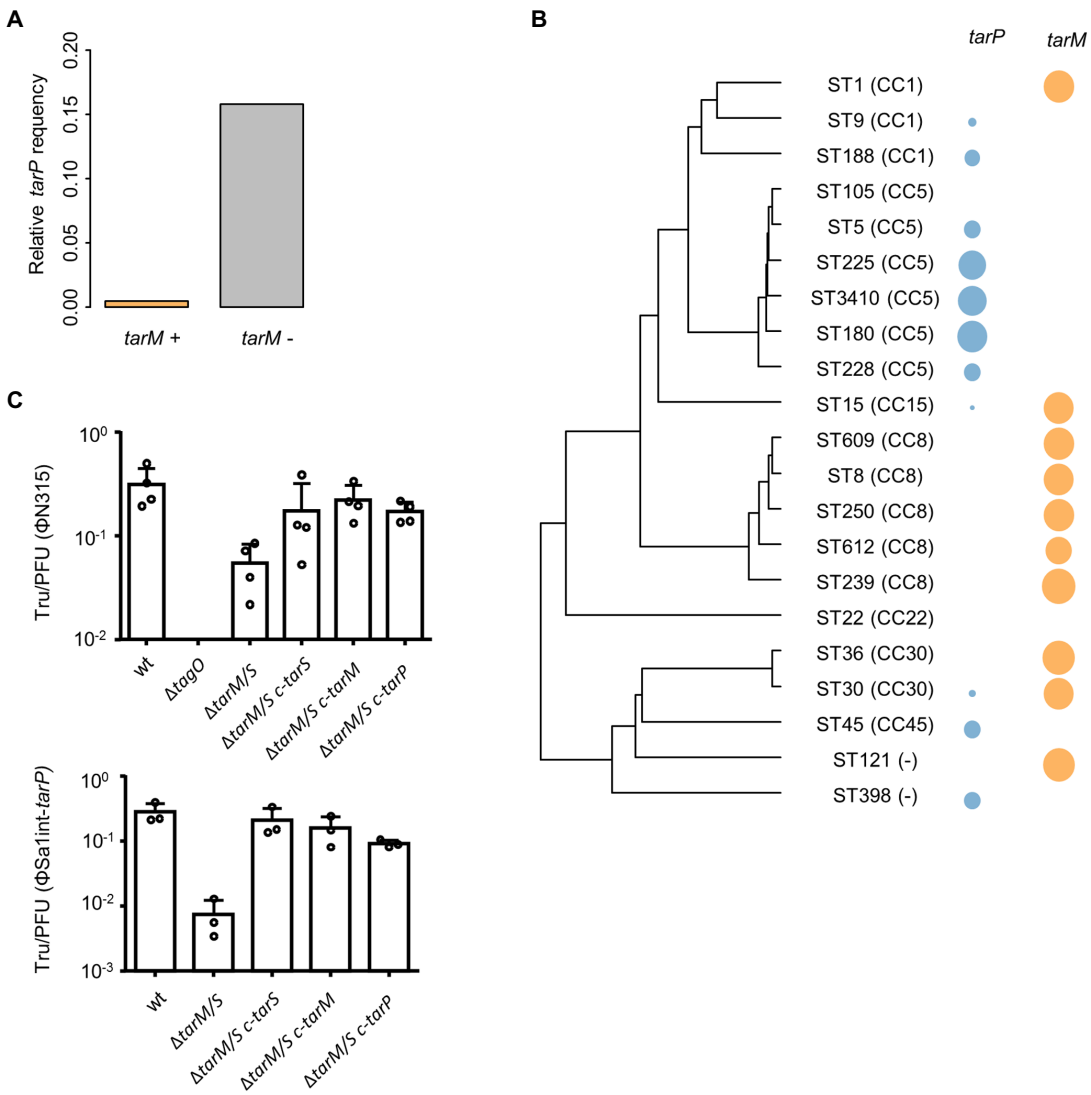
Influence of *tarM* on *tarP* phage prevalence. **(A)** Relative frequency of *tarP* presence in in dependence of *tarM* status. **(B)** Phylogeny based on concatenated MLST alleles for most prevalent STs. Relative frequency of *tarP* and *tarM* are indicated by colored circles. **(C)** Transfer rates of *tarP* phages into RN4420 WTA mutant panel. RN4220 WT, RN4220 WTA mutant (Δ*tagO*), and WTA glycosylation mutant RN4220 (Δ*tarM*/*tarS*) expressing different WTA GTs. Transfer rates are represented as obtained transductants per input PFU of the respective phage. Top, transfer of phage ΦN315 (mean +  s.d. of 4 independent experiments). Bottom, transfer of phage ΦSa1int-*tarP* (mean + s.d. of 3 independent biological replicates).

Next, we probed whether a lack of phage receptor compatibility, a major necessity for phage transduction (Winstel et al., 2013; Li et al., 2015; Gerlach et al., 2018), might interfere with spread of *tarP* phages to other clades. Recipients of phage transduction need to carry appropriate phage receptor structures (Winstel et al., 2013). To study if clonal lineages lacking *tarP* may have altered phage receptor, we probed the glycosyltransferes repertoire of those lineages. Doing so we observed a strong negative association of *tarP* with the second accessory WTA GT genes *tarM* among *S. aureus* genomes (*p* < 2.2 × 10^−16^, Fisher’s exact test) (Figure 3A) in a sequence type-dependent manner (Figure 3B). A decrease in lysogenization by ΦSa3int-*tarP* and ΦSa1int-*tarP* of a mutant lacking any WTA glycosylation (RN4220 Δ*tarM*/*tarS*) indicated a preference but no absolute dependence of *tarP* phages for binding glycosylated WTA (Figure 3C). Restoration of glycosylation by expression of either TarM, TarP, or TarS led to similar levels of transduction, which indicated that TarM glycosylation does not directly prevent adsorption of *tarP* phages and cannot explain the lineage-dependent presence or absence of *tarP* prophages.

**Figure 4:**
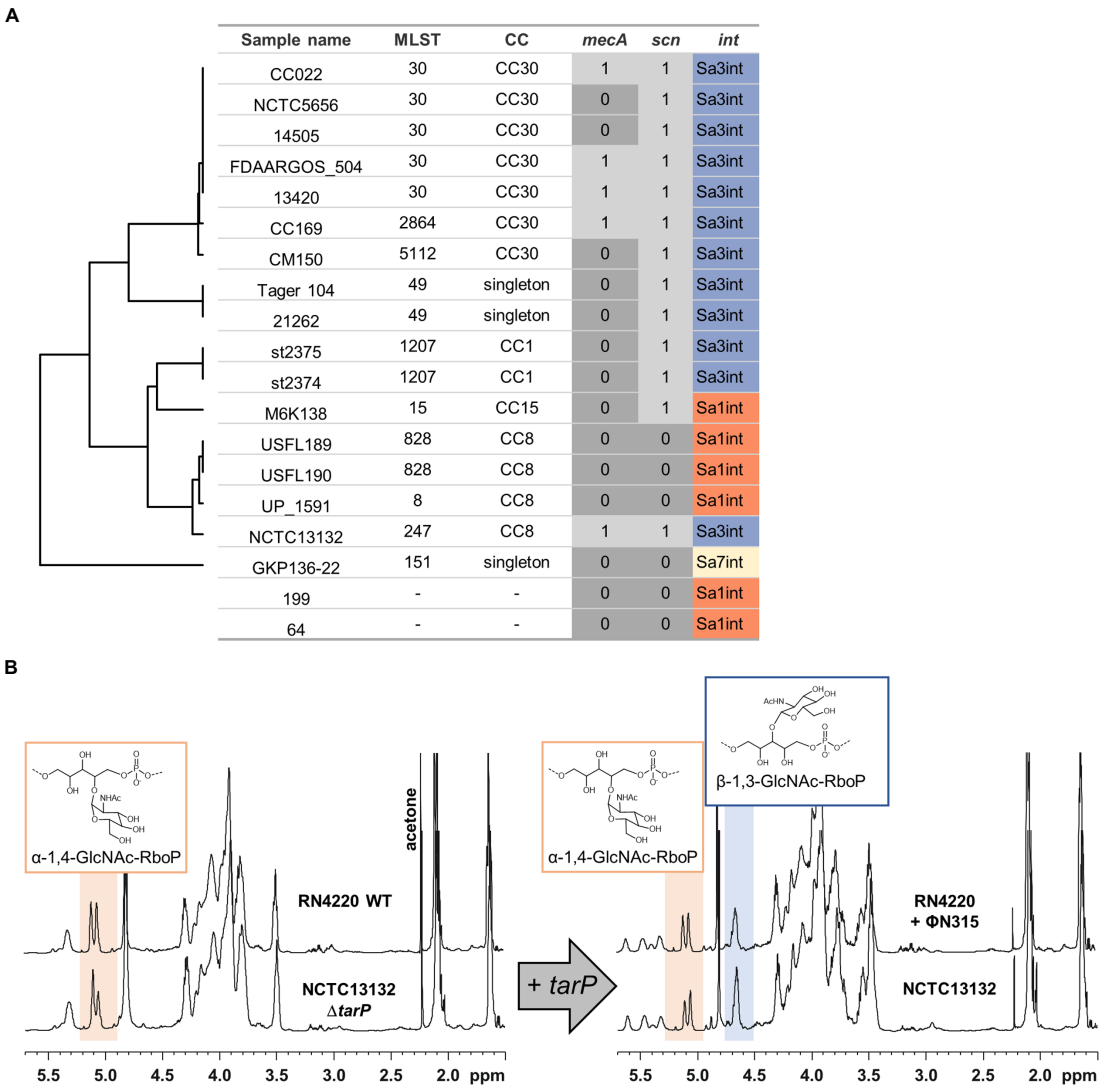
Identified *S. aureus* encoding *tarP* and *tarM*. MRSA determinant *mecA*, IEC, and *tarP*-associated integrase are indicated. **(A)** Isolates are grouped by MLST similarity as in Figure 3B (in 2 cases MLST could not be determined). **(B)** 1H-NMR spectra of *tarM*-positive *S. aureus* without (left) or with *tarP* (right), switching WTA glycosylation from a TarM-type (α-1,4-GlcNAc-RboP) to a TarM/TarP-type (α-1,4-GlcNAc-RboP + β-1,3-GlcNAc-RboP).

The strong negative association of *tarM* and *tarP* was analyzed in more detail to understand, if the two GT might interfere with each other functionally. We searched for naturally occurring *tarM*-encoding, *tarP* phages lysogenic strains in public genomic datasets: of the 11,984 available *S. aureus* genomes we identified only 21, mostly from CC8 and CC30, encoding all three GTs, the accessory genes *tarM*, *tarP,* as well as the housekeeping GT gene *tarS* (Figure 4A). Next, we confirmed the proper glycosylation capability of the engineered *tarP* phages, by utilizing a special set of podoviridae, which depend exclusively on a TarS-conferred 1,4-beta-GlcNAc WTA pattern (Li et al., 2015). These podophages failed to infect the initially sensitive *S*. *aureus* isolates after lysogenization by the engineered *tarP* phages, indicating a successful conversion of the phage receptor by TarP (Supplementary Figure 2A). We used one of these phages, ΦN315, to lysogenize *tarM*-encoding RN4220 and compared the WTA structure with the native lysogen NCTC13132 (CC8) (Figure 4B) using nuclear magnetic resonance (NMR) spectroscopy. All NMR-analyzed strains showed similar growth behavior (Supplementary Figure 2B). TarM-mediated glycosylation was found to be dominant over that conferred by TarS in the *tarP*-negative strains RN4220 and NCTCT13132 Δ*tarP* (Figure 4B left and Table 2), which reflects our previous findings (Xia et al., 2010). In contrast, the two strains additionally expressing *tarP* from the ΦN315 prophage showed signals for both, α-1,4-GlcNAc-RboP and β-1,3-GlcNAc-RboP (conferred by TarM and TarP, respectively) with similar frequency (48 vs. 52% for RN4420 and 40 vs. 60% for NCTC13132) (Figure 4B; Table 2) indicating that TarP and TaM compete with similar efficacy for RboP glycosylation sites and do not exclude each other. To our knowledge this represents the first description of *S. aureus* isolates expressing a novel α-1,4-GlcNAc-RboP -β-1,3-GlcNAc-RboP-WTA mix type, whose biological impact could be of future interest.

**Figure 5:**
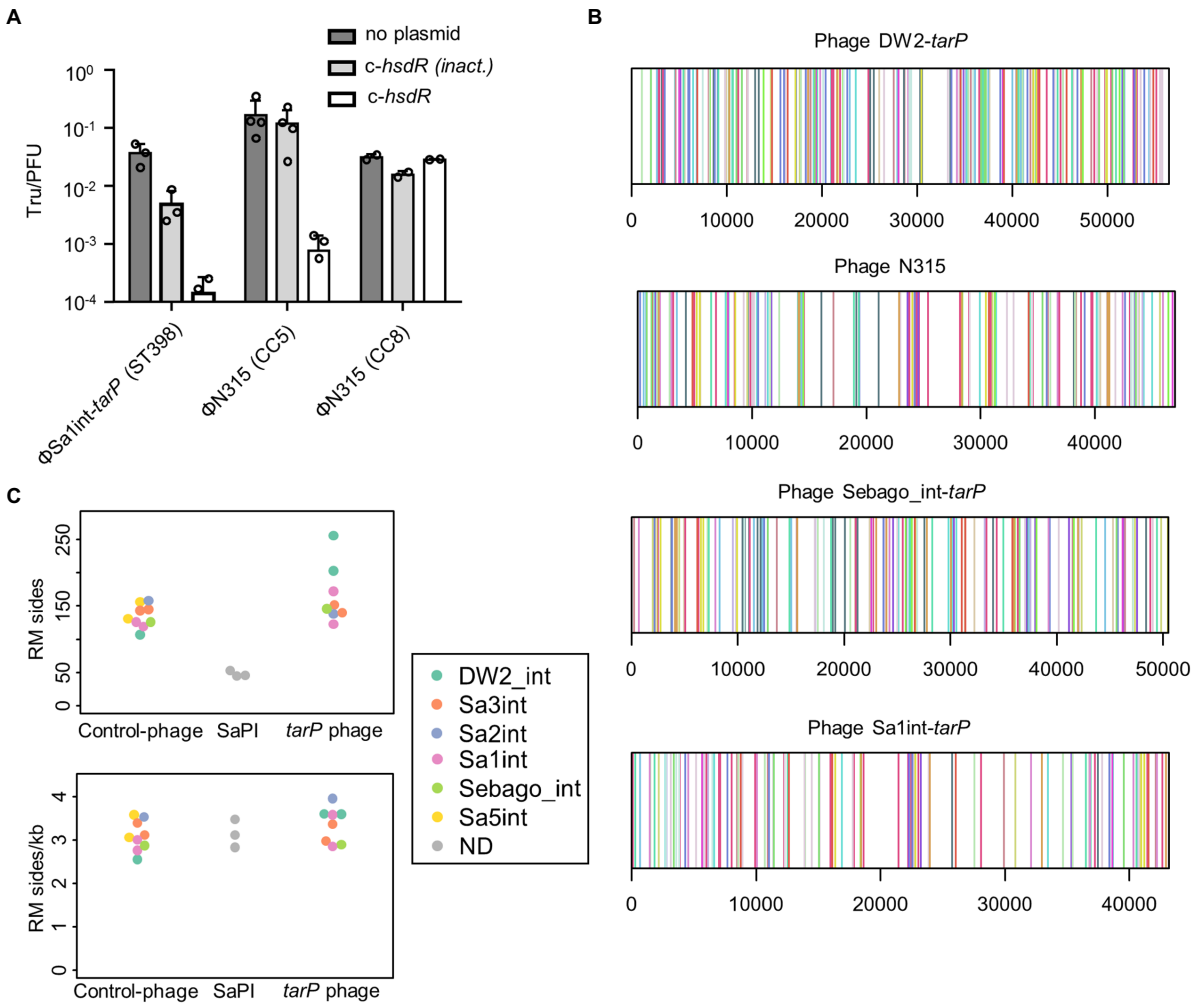
Influence of Type I RM systems on *tarP* phage mobility. **(A)** Transfer of ΦSa1int-*tarP* induced from ST398 background or ΦN315, induced from CC5 or CC8 background, into restriction deficient strain RN4220 (CC8) expressing no plasmid, a control plasmid with the native inactivating *hsdR* mutation (576G>A) of RN4220 or a functional *hsdR* variant from a CC8 isolate (mean Tru/PFU + s.d. of 2 or 3 independent replicates). **(B)** Genetic location of 21 different RM recognition sites (indicated by color) in 4 representative *tarP* phages of the most prevalent *tarP* phage *int* classes, including phages used for *in-vitro* studies. **(C)** Comparison of the sum of RM recognition sites between *tarP* phages, non-*tarP* phages and phage-like SaPIs. The int group for phages is indicated. Top, absolute number of recognition sides. Bottom relative frequency given as sites per 1 kb.

**Table 2:** Relative abundance of NMR motifs for analyzed tarM-encoding *tarP* phage lysogens (lab-made RN4220 ΦN315 and native NCTC13132) and the corresponding strains without *tarP* locus (RN4420 and NCTC13132 Δ*tarP*).

Sample	Total GlcNAc vs. total Rbo [%]	α-GlcNAc vs. total Rbo [%]	β-GlcNAc vs. total Rbo [%]	α-GlcNAc vs. total GlcNAc [%]	% β-GlcNAc vs. total GlcNAc
RN4220 ΦN315	78	37	40	48	52
NCTC13132	83	33	50	40	60
RN4220	85	85	0	100	0
NCTC13132 Δ*tarP*	91	91	0	100	0

### Clone-specific RM systems restrict the transfer of *tarP* phages

**Figure 6:**
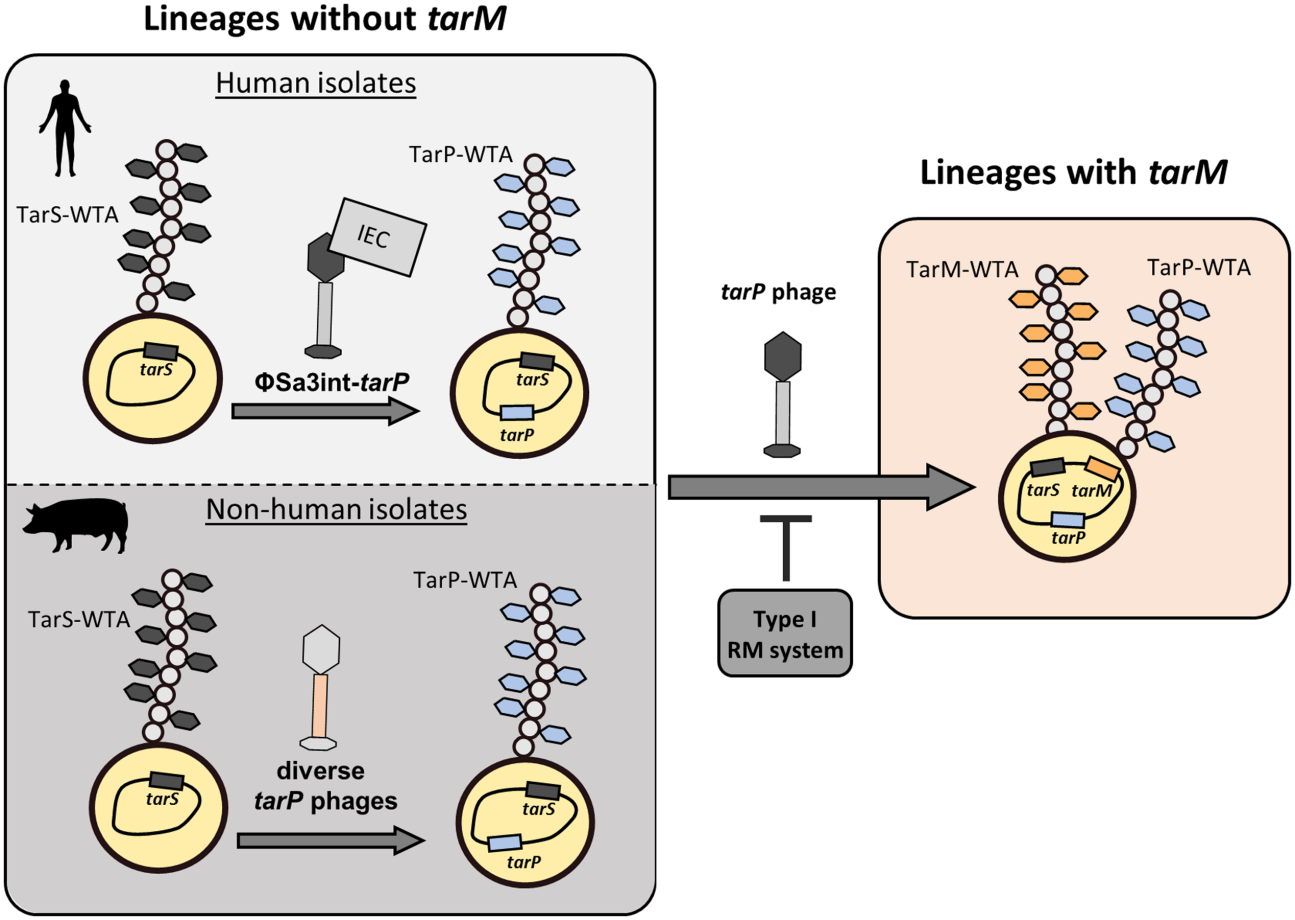
Summary of distribution of *tar* phages. *tarP* phages are prolific in clonal lineages of *S. aureus* that are devoid of the genome-encoded, alternative glycosyltransferase *tarM*. Here, TarP-glycosylation appears to be dominant over TarS-glycosylation. Furthermore, the make-up of *tarP* phages shows a stark discrepancy between Sa3int-phages with presumed IEC in human isolates and diverse non-Sa3int-phages in non-human isolates. Upon integration of *tarP* phages, lysogens express a dominant *tarP*-WTA. Transfer of *tarP* phages into *tarM*-encoding clonal lineages such as (CC1, CC8, and CC30) is mainly suppressed by type I RM systems. However rare bypassing of the host restriction barrier leads to expression of a TarM/TarP-hybrid-WTA.

*Staphylococcus aureus* CCs usually share specific types of lineage-specific RM systems, which might explain the clonal distribution of *tarP phages* (Lee et al., 2019). *S. aureus* mutants lacking Type I RM systems were used to assess this hypothesis. ΦN315 could not lysogenize USA300 JE2 (CC8) but it could be transferred effectively to an isogenic mutant with inactivated *hsdR* locus (Figure 2A). This also demonstrates that *tarP* phages appear not to be affected by prophage immunity, since USA300 harbours a non-*tarP* Sa3int prophage. Overall recipients could be lysogenized by ΦN315 with similar efficacy when they shared the clonal background of the donor strain (Figures 2A,B), which is in agreement with a crucial role of RM systems in susceptibility to *tarP* phage lysogenization. In a second experiment, reminiscent of previous work by Waldron and Lindsay (Waldron and Lindsay, 2006), we reintroduced a functional copy of *sauI* (*hsdR*) from another CC8 isolate into RN4220 (CC8) to restore its Type I RM functionality, which resulted in a profound decrease of lysogenization efficiency for *tarP* phages Sa1int and Sa3int (ΦN315) (Figure 6A). Moreover, transferring ΦSa3int-*tarP* released from a donor strain (USA300) to RN4420 with the same CC8-specific DNA methylation pattern resulted in very similar transfer efficiencies (Figure 6A).

To assess the potential of HsdR proteins to cleave prophage DNA we searched for selected recognition sequences of published SauI RM systems (Cooper et al., 2017) in *tarP* phage genomes. All probed *tarP* phages contained at least one target sequence of each of the 21 known HsdS types. The recognition sites were spread over the entire phage genomes (Figure 3A) implying potentially successful restriction and interception of the infection process by the various HdsS variants. *tarP* phages showed similar frequencies of *S. aureus* HdsS recognition sites compared to control siphophages or phage-related *S. aureus* pathogenicity islands (SaPI), which are devoid of the *tarP* gene (Figure 6C). Thus, distribution of *tarP* phages in other clonal lineages and their dissemination appears to be similarly limited by Type I RM systems as reported for other temperate phages (McCarthy and Lindsay, 2012).

*tarP* phages were abundant in all STs of CC5 besides ST105 (Table 1), which is in agreement with the shared RM systems in this CC (Lee et al., 2019). CC5 and CC8 both use the Newman_MS1 HsdS variant (Lee et al., 2019), which may be a reason why some CC8 isolates also harbor *tarP* phages (Table 1). However, the two clonal groups have different additional RM systems and CC8 encodes *tarM* in its core genome, which may explain why *tarP* has remained rare in CC8. *tarP* is prevalent in CC5, CC398, and CC45 (Table 1) although these clones have quite different RM systems (Lee et al., 2019), indicating that *tarP* phages can expand in different clonal backgrounds once they have traversed a RM barrier. Fortunately, such host clone jumps may have remained rare because *tarP* was not found in any of the genomes from several other major *S. aureus* clonal groups.

## Discussion

*Staphylococcus aureus* is a quickly evolving pathogen, with new clonal lineages continuously emerging and outcompeting existing clones. It is a major concern that HGT will lead to new combinations of virulence, fitness, and resistance factors, creating new clones with even more dangerous properties, than the existing clones (Lee et al., 2018). The *tarP* gene is an *S. aureus* virulence factor of concern as it resides on an MGE and provides host bacteria with extraordinary immune evasion capacities (Gerlach et al., 2018). It has remained enigmatic, why it is found only in a narrow subset of *S. aureus* clonal lineages.

We provide evidence that *tarP* phages are indeed mobile and can lysogenize susceptible *S. aureus* host cells. Intriguingly, the presence of *tarP* was strongly associated with the absence of the alternative GT gene *tarM*, raising the possibility that *tarM*-mediated WTA modification might interfere with infection by *tarP* phages. However, we found that TarM-glycosylation has no impact on *S. aureus* susceptibility to *tarP* phages, while the major factors limiting the spread of *tarP* phages to distant clonal lineages turned out to be RM systems (Figure 6). In a species wide analysis we detected a low frequency of *tarM*-encoding isolates that show lysogenization by tarP phages, which led to the production of a novel TarM/TarP-hybrid WTA. The low frequency of *tarP* phages in *tarM*-encoding *S. aureus* lineages was corroborated also by a recent study (Xiong et al., 2020). Using PCR typing, the authors identified only 2 out of 555 *S. aureus* isolates encoding both, *tarP* and *tarM*.

The fact that *tarP* is also found in LA isolates, localized on non-Sa3int phages, points towards an important role of TarP glycosylation also in non-human hosts, as reported previously (Sieber et al., 2020). However, the obvious preference of *tarP* for Sa3int-phages, might be driven by complex coevolution events of *tarP*, IEC, and the phage itself.

TarP and TarM have similar impacts on many different physiological processes such as resistance to podophages (i.e., 44AHJD-like podophages) (Li et al., 2015; Gerlach et al., 2018) or reduced recognition by host IgGs (van Dalen et al., 2019, 2020). This indicates that *tarP* phages may be particularly successful in providing additional fitness to clonal lineages, which have lost the *tarM* locus and only produce TarS-glycosylated WTA. Here TarP glycosylation appears to become dominant over TarS glycosylation upon lysogenization by the respective prophage (Supplementary Figure 2A). *tarP* prophages would allow their hosts to modulate the immune and phage evasion capacities. A recent study pointed towards similar capabilities of TarS- and TarP-WTA to bind the skin dendritic cell receptor langerin. However, the consequences of TarP and TarS-mediated WTA modification are not identical in terms of immune activation, since *tarP*-expressing *S. aureus* showed increased levels of IL-8 and TNFα secretion by Langerhans cells compared to those with TarS-WTA (Hendriks et al., 2021).

*tarP* phages were present in two *S. aureus* lineages (ST5 and CC1) in a manner that appeared to result from horizontal spread among different phylogenetic backgrounds, while *tarP* distribution patterns in other lineages were in line with those expected under vertical inheritance. In support of a prominent role of restriction mechanisms in suppression of trans-lineage spread of prophages, we found similar abundances of Type-I RM targets in phages with or without *tarP*, supporting the notion that siphophages including *tarP* phages show a broad specificity for RboP WTA receptors with GlcNAc, irrespective of the glycosylation pattern, but are impeded strongly by host restriction (Figure 6). Once *tarP* phages form infectious particles, they are not hindered by WTA glycosylation configurations to lysogenize host strains.

Genotypic risk assessment strategies for *S. aureus* have previously focused strongly on the presence of antibiotic resistance or toxins genes associated with disease severity (Shopsin and Kreiswirth, 2001). Our increasing knowledge of prevalence, function, and phenotypes of WTA GTs may lead to additional suitable biomarkers for monitoring *S. aureus* immune evasion capacities. Understanding the capacities of resistance genes to spread among *S. aureus* strains will be important for estimating their potential contribution to the evolution of new clonal lineages. In this regard, the high mobility of *tarP* remains a matter of concern despite its partial restriction by RM systems.

## Data availability statement

The raw data supporting the conclusions of this article will be made available by the authors, without undue reservation.

## Author contributions

DG, JK, and JB performed phage experiments, WTA extractions, and molecular cloning. CC and AM analyzed WTA *via* NMR. RS, DG, and JL performed bioinformatic analysis of *Staphylococcus aureus* genomes. DG and AP analyzed the data and wrote the manuscript. All authors contributed to the article and approved the submitted version.

## Funding

This work was financed by grants from the Deutsche Forschungsgemeinschaft, grants TRR34, SPP 2330, TRR261 project ID 398967434, and PE 805/7-1 to AP; and the German Center of Infection Research to AP. AP was supported by infrastructural funding from the Cluster of Excellence EXC 2124 “Controlling Microbes to Fight Infections” project ID 390838134.

## Conflict of interest

The authors declare that the research was conducted in the absence of any commercial or financial relationships that could be construed as a potential conflict of interest.

## Publisher’s note

All claims expressed in this article are solely those of the authors and do not necessarily represent those of their affiliated organizations, or those of the publisher, the editors and the reviewers. Any product that may be evaluated in this article, or claim that may be made by its manufacturer, is not guaranteed or endorsed by the publisher.

## Footnotes

^1^ https://github.com/tseemann/mlst

## References

[ref1] Altschul S. F. Gish W. Miller W. Myers E. W. Lipman D. J. (1990). Basic local alignment search tool. J. Mol. Biol. 215, 403–410. doi: 10.1016/S0022-2836(05)80360-2 2231712

[ref2] Brown S. Santa Maria J. P. Jr. Walker S. (2013). Wall teichoic acids of gram-positive bacteria. Annu. Rev. Microbiol. 67, 313–336. doi: 10.1146/annurev-micro-092412-155620, PMID: 24024634 PMC3883102

[ref3] Brown S. Xia G. Luhachack L. G. Campbell J. Meredith T. C. Chen C.. (2012). Methicillin resistance in *Staphylococcus aureus* requires glycosylated wall teichoic acids. Proc. Natl. Acad. Sci. U. S. A. 109, 18909–18914. doi: 10.1073/pnas.1209126109, PMID: 23027967 PMC3503181

[ref4] Chen P. S. T. Y. Warner H. (1956). Microdetermination of phosphorus. Anal. Chem. 28, 1756–1758. doi: 10.1021/ac60119a033

[ref5] Cooper L. P. Roberts G. A. White J. H. Luyten Y. A. Bower E. K. M. Morgan R. D.. (2017). DNA target recognition domains in the type I restriction and modification systems of *Staphylococcus aureus*. Nucleic Acids Res. 45, 3395–3406. doi: 10.1093/nar/gkx067, PMID: 28180279 PMC5399793

[ref6] Croucher N. J. Page A. J. Connor T. R. Delaney A. J. Keane J. A. Bentley S. D.. (2015). Rapid phylogenetic analysis of large samples of recombinant bacterial whole genome sequences using Gubbins. Nucleic Acids Res. 43:e15. doi: 10.1093/nar/gku1196, PMID: 25414349 PMC4330336

[ref7] Geiger T. Francois P. Liebeke M. Fraunholz M. Goerke C. Krismer B.. (2012). The stringent response of *Staphylococcus aureus* and its impact on survival after phagocytosis through the induction of intracellular PSMs expression. PLoS Pathog. 8:e1003016. doi: 10.1371/journal.ppat.1003016, PMID: 23209405 PMC3510239

[ref8] Gerlach D. Guo Y. De Castro C. Kim S. H. Schlatterer K. Xu F. F.. (2018). Methicillin-resistant *Staphylococcus aureus* alters cell wall glycosylation to evade immunity. Nature 563, 705–709. doi: 10.1038/s41586-018-0730-x, PMID: 30464342

[ref9] Goerke C. Pantucek R. Holtfreter S. Schulte B. Zink M. Grumann D.. (2009). Diversity of prophages in dominant *Staphylococcus aureus* clonal lineages. J. Bacteriol. 191, 3462–3468. doi: 10.1128/JB.01804-08, PMID: 19329640 PMC2681900

[ref10] Hendriks A. van Dalen R. Ali S. Gerlach D. van der Marel G. A. Fuchsberger F. F.. (2021). Impact of glycan linkage to *Staphylococcus aureus* wall teichoic acid on Langerin recognition and Langerhans cell activation. ACS Infect. Dis. 7, 624–635. doi: 10.1021/acsinfecdis.0c00822, PMID: 33591717 PMC8023653

[ref11] Ingmer H. Gerlach D. Wolz C. (2019). Temperate phages of *Staphylococcus aureus*. Microbiol. Spectr 7. doi: 10.1128/microbiolspec.GPP3-0058-2018, PMID: 31562736 PMC10921950

[ref12] Jolley K. A. Maiden M. C. (2010). BIGSdb: scalable analysis of bacterial genome variation at the population level. BMC Bioinformatics 11:595. doi: 10.1186/1471-2105-11-595, PMID: 21143983 PMC3004885

[ref13] Klotz K. Korn A. Newkirk H. Liu M. Gill J. J. Ramsey J. (2019). Complete genome sequence of *Staphylococcus aureus* Siphophage Sebago. Microbiol. Resour. Announc. 8. doi: 10.1128/MRA.00765-19, PMID: 31320442 PMC6639632

[ref14] Lee J. Y. H. Carter G. P. Pidot S. J. Guérillot R. Seemann T. Gonçalves da Silva A.. (2019). Mining the methylome reveals extensive diversity in *Staphylococcus epidermidis* restriction modification. MBio 10. doi: 10.1128/mBio.02451-19, PMID: 31848274 PMC6918075

[ref15] Lee A. S. de Lencastre H. Garau J. Kluytmans J. Malhotra-Kumar S. Peschel A.. (2018). Methicillin-resistant *Staphylococcus aureus*. Nat. Rev. Dis. Primers. 4:18033. doi: 10.1038/nrdp.2018.33 29849094

[ref16] Li X. Gerlach D. Du X. Larsen J. Stegger M. Kuhner P.. (2015). An accessory wall teichoic acid glycosyltransferase protects *Staphylococcus aureus* from the lytic activity of podoviridae. Sci. Rep. 5:17219. doi: 10.1038/srep17219, PMID: 26596631 PMC4667565

[ref17] Lindsay J. A. (2010). Genomic variation and evolution of *Staphylococcus aureus*. Int. J. Med. Microbiol. 300, 98–103. doi: 10.1016/j.ijmm.2009.08.013 19811948

[ref18] Maslanova I. Doskar J. Varga M. Kuntova L. Muzik J. Maluskova D.. (2013). Bacteriophages of *Staphylococcus aureus* efficiently package various bacterial genes and mobile genetic elements including SCCmec with different frequencies. Environ. Microbiol. Rep. 5, 66–73. doi: 10.1111/j.1758-2229.2012.00378.x, PMID: 23757132

[ref19] McCarthy A. J. Lindsay J. A. (2012). The distribution of plasmids that carry virulence and resistance genes in *Staphylococcus aureus* is lineage associated. BMC Microbiol. 12:104. doi: 10.1186/1471-2180-12-104, PMID: 22691167 PMC3406946

[ref20] Monk I. R. Tree J. J. Howden B. P. Stinear T. P. Foster T. J. (2015). Complete bypass of restriction Systems for Major *Staphylococcus aureus* lineages. MBio 6, e00308–e00315. doi: 10.1128/mBio.00308-15, PMID: 26015493 PMC4447248

[ref21] Nguyen L. T. Schmidt H. A. von Haeseler A. Minh B. Q. (2015). IQ-TREE: a fast and effective stochastic algorithm for estimating maximum-likelihood phylogenies. Mol. Biol. Evol. 32, 268–274. doi: 10.1093/molbev/msu300, PMID: 25371430 PMC4271533

[ref22] O’Leary N. A. Wright M. W. Brister J. R. Ciufo S. Haddad D. McVeigh R.. (2016). Reference sequence (RefSeq) database at NCBI: current status, taxonomic expansion, and functional annotation. Nucleic Acids Res. 44, D733–D745. doi: 10.1093/nar/gkv1189, PMID: 26553804 PMC4702849

[ref23] Oliveira P. H. Touchon M. Rocha E. P. (2014). The interplay of restriction-modification systems with mobile genetic elements and their prokaryotic hosts. Nucleic Acids Res. 42, 10618–10631. doi: 10.1093/nar/gku734, PMID: 25120263 PMC4176335

[ref24] Oliveira P. H. Touchon M. Rocha E. P. (2016). Regulation of genetic flux between bacteria by restriction-modification systems. Proc. Natl. Acad. Sci. U. S. A. 113, 5658–5663. doi: 10.1073/pnas.1603257113, PMID: 27140615 PMC4878467

[ref25] Richardson E. J. Bacigalupe R. Harrison E. M. Weinert L. A. Lycett S. Vrieling M.. (2018). Gene exchange drives the ecological success of a multi-host bacterial pathogen. Nat. Ecol. Evol. 2, 1468–1478. doi: 10.1038/s41559-018-0617-0, PMID: 30038246 PMC7610605

[ref26] Sadykov M. R. (2016). Restriction-modification systems as a barrier for genetic manipulation of *Staphylococcus aureus*. Methods Mol. Biol. 1373, 9–23. doi: 10.1007/7651_2014_180, PMID: 25646604

[ref27] Sahl J. W. Lemmer D. Travis J. Schupp J. M. Gillece J. D. Aziz M.. (2016). NASP: an accurate, rapid method for the identification of SNPs in WGS datasets that supports flexible input and output formats. Microb. Genom. 2:e000074. doi: 10.1099/mgen.0.000074, PMID: 28348869 PMC5320593

[ref28] Scharn C. R. Tenover F. C. Goering R. V. (2013). Transduction of staphylococcal cassette chromosome mec elements between strains of *Staphylococcus aureus*. Antimicrob. Agents Chemother. 57, 5233–5238. doi: 10.1128/AAC.01058-13, PMID: 23939891 PMC3811280

[ref29] Sheppard S. K. Guttman D. S. Fitzgerald J. R. (2018). Population genomics of bacterial host adaptation. Nat. Rev. Genet. 19, 549–565. doi: 10.1038/s41576-018-0032-z 29973680

[ref30] Shopsin B. Kreiswirth B. N. (2001). Molecular epidemiology of methicillin-resistant *Staphylococcus aureus*. Emerg. Infect. Dis. 7, 323–326. doi: 10.3201/eid0702.010236, PMID: 11294733 PMC2631714

[ref31] Sieber R. N. Urth T. R. Petersen A. Moller C. H. Price L. B. Skov R. L.. (2020). Phage-mediated immune evasion and transmission of livestock-associated methicillin-resistant *Staphylococcus aureus* in humans. Emerg. Infect. Dis. 26. doi: 10.3201/eid2611.201442, PMID: 33079052 PMC7588543

[ref32] Smith L. R. G. E. (1979). Quantitation of glycosaminoglycan hexosamine using 3-methyl-2-benzothiazolone hydrazone hydrochloride. Anal. Biochem. 98, 478–480. doi: 10.1016/0003-2697(79)90170-2, PMID: 496014

[ref33] Speciale I. Notaro A. Garcia-Vello P. Di Lorenzo F. Armiento S. Molinaro A.. (2022). Liquid-state NMR spectroscopy for complex carbohydrate structural analysis: a hitchhiker's guide. Carbohydr. Polym. 277:118885. doi: 10.1016/j.carbpol.2021.118885, PMID: 34893288

[ref34] van Dalen R. Molendijk M. M. Ali S. van Kessel K. P. M. Aerts P. van Strijp J. A. G.. (2019). Do not discard *Staphylococcus aureus* WTA as a vaccine antigen. Nature 572, E1–E2. doi: 10.1038/s41586-019-1416-8, PMID: 31367020

[ref35] van Dalen R. Peschel A. van Sorge N. M. (2020). Wall teichoic acid in *Staphylococcus aureus* host interaction. Trends Microbiol. 28, 985–998. doi: 10.1016/j.tim.2020.05.017, PMID: 32540314

[ref36] Waldron D. E. Lindsay J. A. (2006). Sau1: a novel lineage-specific type I restriction-modification system that blocks horizontal gene transfer into *Staphylococcus aureus* and between *S. aureus* isolates of different lineages. J. Bacteriol. 188, 5578–5585. doi: 10.1128/JB.00418-06, PMID: 16855248 PMC1540015

[ref37] Weidenmaier C. Peschel A. (2008). Teichoic acids and related cell-wall glycopolymers in gram-positive physiology and host interactions. Nat. Rev. Microbiol. 6, 276–287. doi: 10.1038/nrmicro1861, PMID: 18327271

[ref38] Wilson G. G. Murray N. E. (1991). Restriction and modification systems. Annu. Rev. Genet. 25, 585–627. doi: 10.1146/annurev.ge.25.120191.003101 1812816

[ref39] Winstel V. Liang C. Sanchez-Carballo P. Steglich M. Munar M. Broker B. M.. (2013). Wall teichoic acid structure governs horizontal gene transfer between major bacterial pathogens. Nat. Commun. 4:2345. doi: 10.1038/ncomms3345, PMID: 23965785 PMC3903184

[ref40] Winstel V. Xia G. Peschel A. (2014). Pathways and roles of wall teichoic acid glycosylation in *Staphylococcus aureus*. Int. J. Med. Microbiol. 304, 215–221. doi: 10.1016/j.ijmm.2013.10.009, PMID: 24365646

[ref41] Xia G. Maier L. Sanchez-Carballo P. Li M. Otto M. Holst O.. (2010). Glycosylation of wall teichoic acid in *Staphylococcus aureus* by TarM. J. Biol. Chem. 285, 13405–13415. doi: 10.1074/jbc.M109.096172, PMID: 20185825 PMC2859500

[ref42] Xia G. Wolz C. (2014). Phages of *Staphylococcus aureus* and their impact on host evolution. Infect. Genet. Evol. 21, 593–601. doi: 10.1016/j.meegid.2013.04.022, PMID: 23660485

[ref43] Xiong M. Zhao J. Huang T. Wang W. Wang L. Zhao Z.. (2020). Molecular characteristics, virulence gene and Wall teichoic acid glycosyltransferase profiles of *Staphylococcus aureus*: a multicenter study in China. Front. Microbiol. 11:2013. doi: 10.3389/fmicb.2020.02013, PMID: 32973729 PMC7466653

[ref44] Yu G. Smith D. K. Zhu H. Guan Y. Lam T. T. Y. McInerny G. (2016). Ggtree: an R package for visualization and annotation of phylogenetic trees with their covariates and other associated data. Methods Ecol. Evol. 8, 28–36. doi: 10.1111/2041-210x.12628

