# Supplementary figures and images for: Horizontal transfer and phylogenetic distribution of the immune evasion factor tarP
Source: Front Microbiol. 2022 Oct 28;13:951333. doi: 10.3389/fmicb.2022.951333 (PMC9650247; doi:10.3389/fmicb.2022.951333)

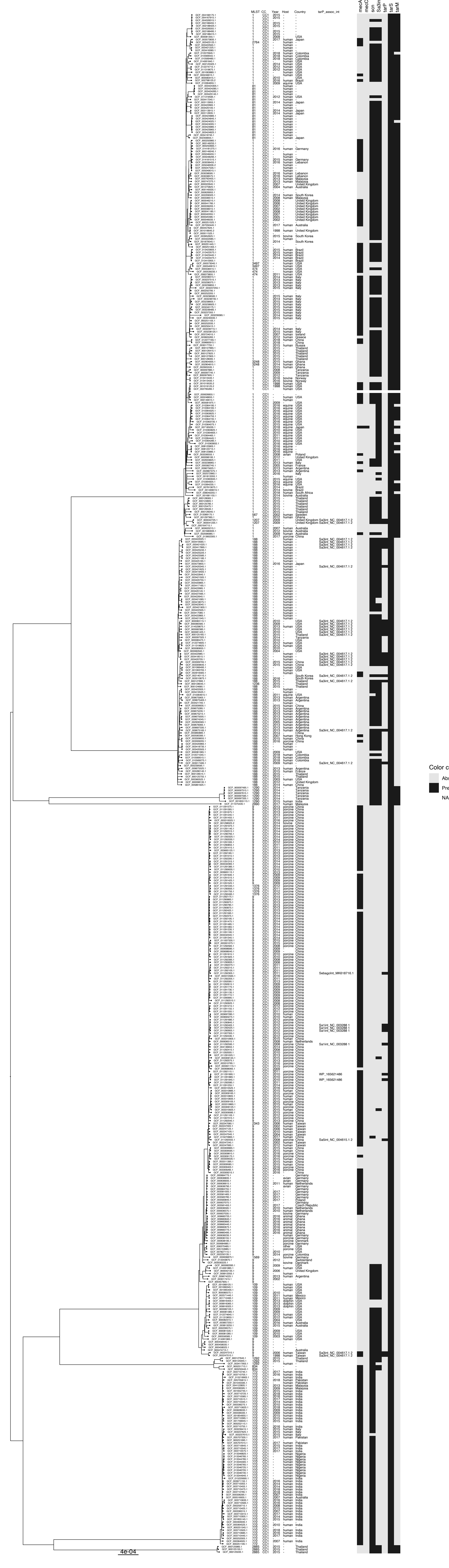

Supplement: Supplementary file 1 [file Data_Sheet_1.PDF]

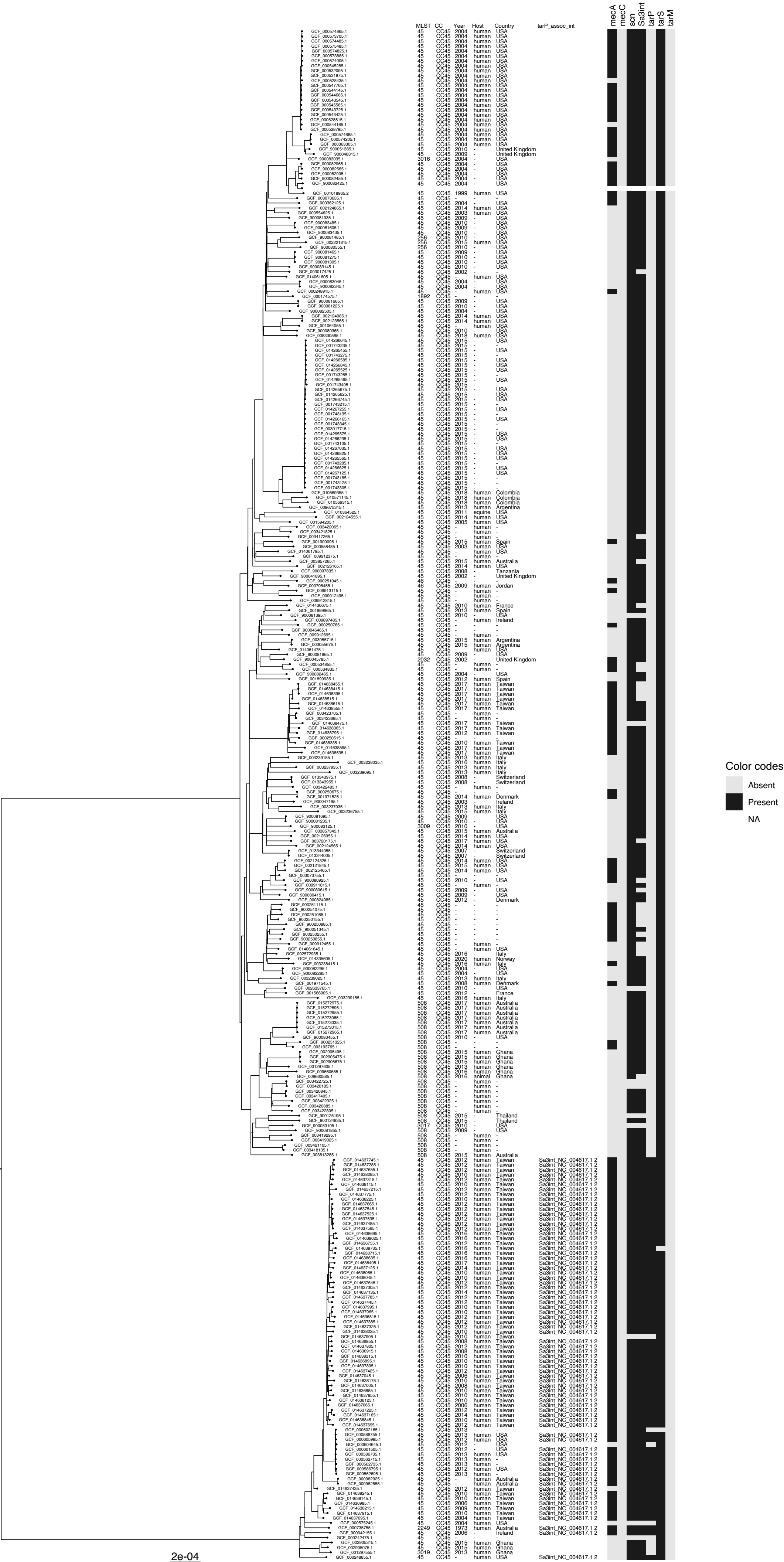

Supplement: Supplementary file 2 [file Data_Sheet_2.PDF]

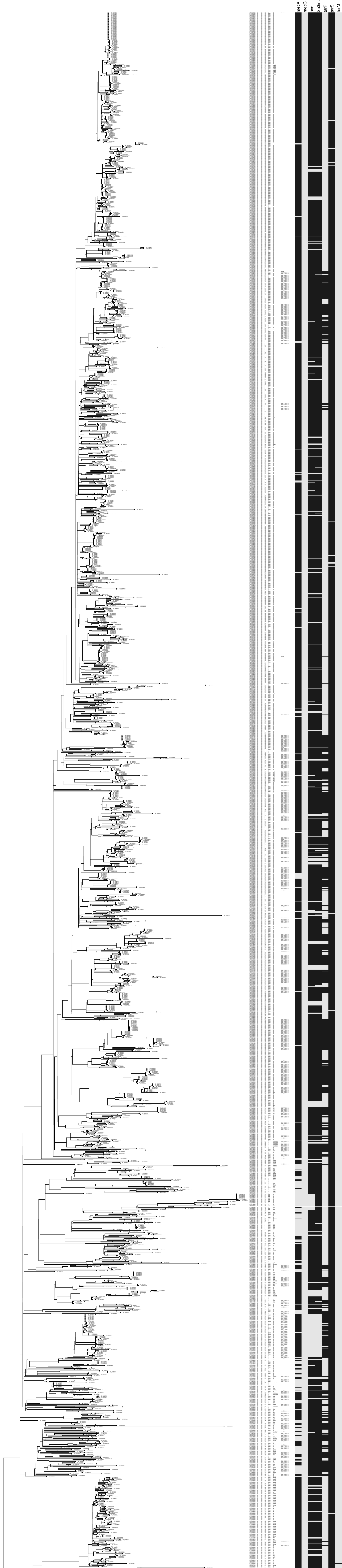

Color codes

Absent

Present

NA

Supplement: Supplementary file 3 [file Data_Sheet_3.PDF]
