# Supplementary material for: Horizontal transfer and phylogenetic distribution of the immune evasion factor tarP
Source: Front Microbiol. 2022 Oct 28;13:951333. doi: 10.3389/fmicb.2022.951333 (PMC9650247; doi:10.3389/fmicb.2022.951333)
Supplement: Supplementary file 4 [file Data_Sheet_4.docx]

Supplementary Material

## Supplementary figures and tables


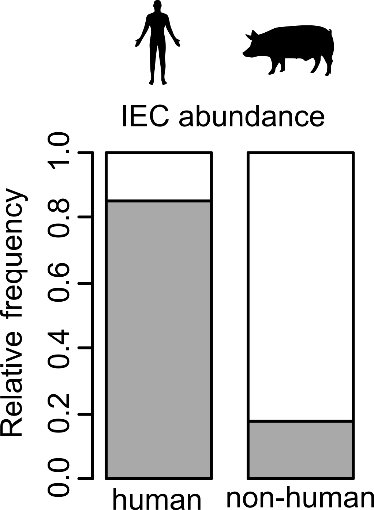


**Supplementary Figure 1.** Relative frequency of genetic marker IEC in dependence of isolate origin.


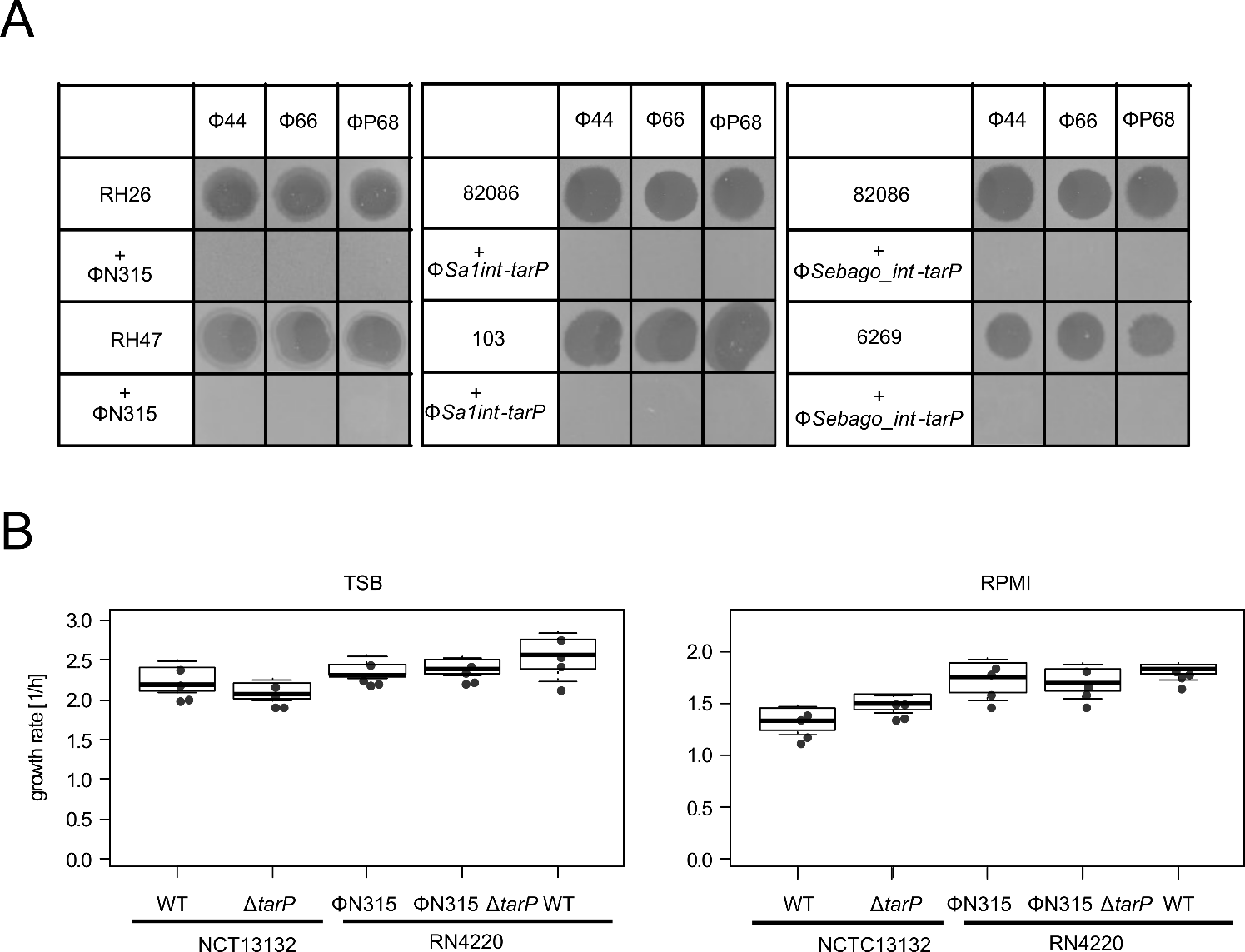


**Supplementary Figure 2.** (A) *tarP* phages ΦN315, ΦSa1int-*tarP*, and ΦSebago_int-*tarP* were transferred into representative *S. aureus* strains (left: CC5, middle and right: CC398) only expressing *tarS*. Resulting lysogens were probed for resistance to podophages (Φ44, Φ66, ΦP68) indicating successful conversion of TarS-WTA to TarP-WTA. (B) Growth rates of *tarM*-encoding strain NCTC13132 and RN4220 lysogenized with ΦN315 in two different media (TSB, RPMI). Growth rates are displayed by boxplots of 4 datapoints from 2 independent biological replicates.


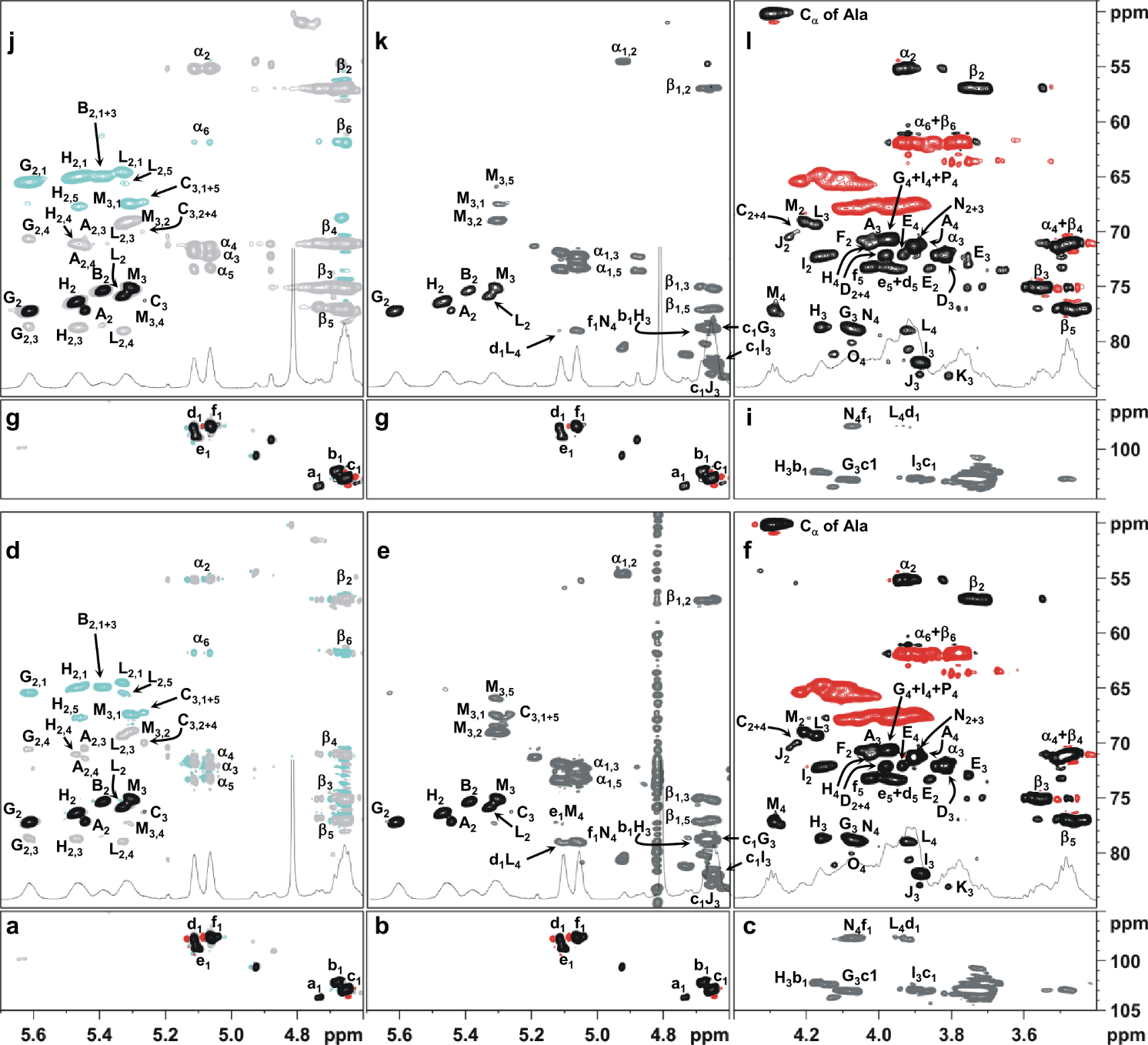


**Supplementary Figure 3.** 2D NMR spectroscopy: y-axes and x-axes show ^13^C and ^1^H chemical shifts**.**  Measurement occurred at 600 MHz, 298 K. NMR spectra acquired for RN4220 ΦN315 (panels a-f), NCTC13132 (panels g-l). In all the spectra, the most relevant densities are labelled with the letter used in Supplementary Table 2, as for the GlcNAc units, the anomeric signals are indicated with a small letter, while the densities of the other carbon atoms are generically denoted with the configuration a or b of the unit and a number, since these overlap. For RN4220 ΦN315: a) and b) region of the HSQC detailing the anomeric signals; c) HMBC expansion detailing the long range correlation between the carbinolic protons and the anomeric signals; d) overlap of HSQC (black) and HSQC-TOCSY (grey and cyan, recorded with 100 ms mixing time) spectra detailing the correlations between the protons of the anomeric region with the carbon signals of the carbinolic region; e) overlap of HSQC (black) and HMBC (dark grey) spectra detailing the correlations between the protons of the anomeric region with the carbon signals of the carbinolic region; f) HSQC spectrum detailing the carbinolic region, the phase of the CH2 densities is opposed to the others and is red. For NCTC13132 spectra: the panels g-l are constructed with the same logic used for a-f.

**Supplementary Table 1.** ^1^H (600 MHz, plain text) and ^13^C (150 MHz, italic text) chemical shifts of WTA structural motifs collected in *S. aureus* through the study of RN4220 ΦN315, and NCTC13132. By convention, C-1 of the ribitol or of glycerol unit is placed at the left of the structural formula; “P” stands for phosphate; a dotted linkage attached to phosphate indicates a phosphodiester linkage, otherwise phosphate is linked as monoester and the chain is truncated; when phosphate is absent, the chain terminates with an alcoholic function. Ribitol units are labelled with a capital letter, GlcNAc units are β unless their α configuration is reported in explicit; all monosaccharides are labelled with a small letter. Where appropriate, the GlcNAc linked to a ribitol is indicated next to it and its label is in brackets.

| **Label** | **Structural motif** | **1** | **2** | **3** | **4** | **5** |
| --- | --- | --- | --- | --- | --- | --- |
| **A** |  | 4.18 (2X) | 5.45 | 4.02 | 3.88 | 4.05; 3.97 |
|  |  | *64.8* | *77.1* | *70.4* | *71.4* | *67.6* |
| **B** |  | 4.11 (2X) | 5.39 | 4.11 (2X) |  |  |
|  |  | *64.9* | *75.3* | *64.9* |  |  |
| **C** |  | 4.05;3.95 | 4.23 | 5.26 | 4.23 | 4.05;3.95 |
|  |  | *67.3* | *69.9* | *76.3* | *69.9* | *67.3* |
| **D** |  | 4.07;3.96 | 3.98 | 3.81 | 3.98 | 4.07;3.96 |
|  |  | *67.8* | *72.1* | *72.5* | *72.1* | *67.8* |
| **E** |  | 3.81;3.66 | 3.86 | 3.75 | 3.94 | 3.95;3.98 |
|  |  | *63.5* | *73.1* | *73.0* | *72.2* | *67.8* |
| **F** |  | 3.89;3.96 | 4.05 | 3.89;3.96 |  |  |
|  |  | *67.3* | *70.8* | *67.3* |  |  |
| **G** |  | 4.22 (2X) | 5.62 | 4.10 | 3.99 | 3.95;4.09 |
| **(c)** |  | *65.4* | *77.0* | *78.4* | *70.4* | *67.2* |
| **H** |  | 4.17;4.09 | 5.47 | 4.16 | 3.98 | ca 4.02^a^ |
| **(b)** |  | *65.0* | *76.3* | *78.6* | *70.8* | *67.6* |
| **I** |  | 4.02;3.92 | 4.17^b^ | 3.89 | 3.98^b^ | 4.02;3.92 |
| **(c)** |  | *67.8* | *72.2* | *81.8* | *70.6* | *67.8* |
| **J** |  | 4.46;4.40 | 4.20 | 3.89 | as I4 | as I5 |
| **(c)** |  | *68.4* | *69.2* | *82.8* |  |  |
| **K^c^** |  | 3.52;3.72 | 3.90 | 3.81 | as I4 | as I5 |
|  |  | *63.5* | *72.0* | *83.0* |  |  |
| **L** |  | Ca 4.16 | 5.33 | 4.17 | 3.92 | ND |
| **(d)** |  | *64.4* | *75.8* | *69.5* | *79.0* | *65.5* |
| **M** |  | ND | 4.21 | 5.31 | 4.29 | 4.07 |
| **(e)** |  | *68.4* | *68.9* | *75.0* | *77.2* | *67.4* |
| **N** |  | 3.97;4.05 | 3.90 | 3.90 | 4.07 | 4.08;4.12 |
| **(f)** |  | *67.9* | *71.3* | *71.3* | *78.9* | *65.7* |
|  | **1** | **2** | **3** | **4** | **5** | **6** |
| **Ala** | -- | 4.28 | 1.61 |  |  |  |
|  | ND^d^ | *50.3* | *16.4* |  |  |  |
| **a** | 4.73 | 3.76 | 3.56 | 3.50 | 3.47 | 3.94; 3.78 |
| **β-GlcNAc** | *102.5* | *56.8* | *75.1* | *71.0* | *77.0* | *61.8* |
| **b** | 4.68 | 3.75 | 3.54 | 3.48 | 3.48 | 3.94;3.77 |
| **β-GlcNAc** | *103.0* | *56.8* | *75.0* | *70.9* | *77.0* | *61.6* |
| **c** | 4.65 | 3.71 | 3.58 | 3.48 | 3.43 | 3.94;3.77 |
| **β-GlcNAc** | *102.1* | *56.8* | *74.9* | *70.9* | *76.9* | *61.6* |
| **d** | 5.11 | 3.94 | 3.81 | 3.50 | 3.95 | as f6 |
| **α-GlcNAc** | *97.9* | *55.2* | *71.8* | *71.3* | *73.4* |  |
| **e** | 5.11 | 3.94 | 3.81 | 3.50 | 3.97 | as f6 |
| **α-GlcNAc** | *98.7* | *55.2* | *71.8* | *71.3* | *73.4* |  |
| **f** | 5.06 | 3.94 | 3.83 | 3.50 | 4.03 | 3.79;3.90 |
| **α-GlcNAc** | *97.7* | *55.1* | *72.2* | *71.4* | *73.2* | *61.8* |

^a^ Proton chemical shifts difficult to assign due to crowding of signals.

^b^ Attribution can be exchanged

^c^ The identity of the GlcNAc at this position could not be defined with certainty, except that it belongs to the group of the β-configured units.

^d^ In the HMBC, the H-2 of this unit correlated with more than a carbonyl group suggesting the presence of different types of Ala units, in agreement with the finding of several motifs containing this decoration.

**Supplementary Table 2**: composition as motif % of the WTA from *S. aureus* RN4220 ΦN315 and NTCT13132, as calculated by integration of the appropriate HSQC densities. The structural pattern of each motif is detailed in Supplementary Table 1.

| **Motif (NMR code)** | **RN4220 ΦN315%** | **NCTC13132 %** |
| --- | --- | --- |
| **A** | 3.9 | 2.0 |
| **B** | 6.9 | 4.45 |
| **C** | 0.7 | 0.0 |
| **D** | 11.4 | 10.8 |
| **E** | 3.3 | 3.0 |
| **F** | 7.3 | 4.2 |
| **G** | 9.4 | 10.6 |
| **H** | 8.5 | 10.0 |
| **I** | 14.1 | 20.5 |
| **J** | 1.2 | 2.0 |
| **S** | 1.2 | 2.2 |
| **L** | 5.6 | 4.6 |
| **M** | 9.9 | 7.7 |
| **N** | 16.6 | 18.0 |
| **% total Rbo** | 85.7 | 91.3 |
| **% total GlcNAc vs total Rbo** | 77.6 | 82.7 |
| **% α-GlcNAc vs total Rbo** | 37.4 | 33.1 |
| **% β-GlcNAc vs total Rbo** | 40.1 | 49.6 |
| **% α-GlcNAc vs total GlcNAc** | 48.2 | 40.0 |
| **% β-GlcNAc vs total GlcNAc** | 51.7 | 59.9 |

**Supplementary Table 3**: Bacterial strains presented in this study

| **Bacerial strain** | **Information (Sequence type, clonal complex)** | **Reference** |
| --- | --- | --- |
| *S. aureus* N315 WT | φSa3int-*tarP*, ST5, CC5 | (Kuroda et al., 2001) |
| *S. aureus* N315 ΦSa3int-*tarP*::tet | tetM-labelled *tarP phage*, ST5, CC5 | This study |
| *S. aureus* N315 Δ*tarP* ΦSa3int-*tarP*::tet | tetM-labelled *tarP* phage (lacking the tarP locus), ST5, CC5 | This study |
| *S. aureus* 75049 Φ*tarP*-Sa1int::erm | ermB-labelled *tarP phage*, ST398 | This study |
| *S. aureus* RN4220 WT | Restriction deficient, ST8 | (Kreiswirth et al., 1983) |
| *S. aureus* RN4220 Δ*tarM*Δ*tarS* | WTA-glycosylation deficient, ST8 | (Brown et al., 2012) |
| *S. aureus* RN4220 Δ*tarM*Δ*tarS* pRB474*-tarS* | Expression of TarS-WTA | (Brown et al., 2012) |
| *S. aureus* RN4220 Δ*tarM*Δ*tarS* pRB474*-tarM* | Expression of TarM-WTA | (Brown et al., 2012) |
| *S. aureus* RN4220 Δ*tarM*Δ*tarS* pRB474*-tarP* | Expression of TarP-WTA | (Gerlach et al., 2018) |
| *S. aureus* RN4220 ΔtagO | tagO::erm, WTA deficient | (Xia et al., 2011) |
| *S. aureus* RN4220 ΦN315 | Lysogen of ΦSa3int-*tarP*::tet | This study |
| *S. aureus* NCTC13132 | ST247, CC8 | Obtained from NCTC collection |
| *S. aureus* NCTC13132 Δ*tarP* | *tarP* mutant | This study |
| *S. aureus* 82086 | ST398, LA-MRSA | (Winstel et al., 2015) |
| *S. aureus* 75049 | ST398 LA-MRSA | Jesper Larsen, SSI |
| *S. aureus* 75049 ΦSa1int-*tarP*::erm | *ermB*-labelled *tarP* phage, ST398 | This study |
| *S. aureus* 103 | ST398, LA-MRSA | Statens Serum institute Copenhagen |
| *S. aureus* 105 | ST398, LA-MRSA | Statens Serum institute Copenhagen |
| *S. aureus* 6269 | ST398, LA-MRSA | Statens Serum institute Copenhagen |
| *S. aureus* NRS229 | ST1, CC1 | NARSA collection |
| *S. aureus* Oxford207 | ST15, CC15 | (Enright et al., 2000) |
| *S. aureus* NRS71 | ST30, CC30 | NARSA collection |
| *S. aureus* NRS184 | ST22, CC22 | NARSA collection |
| *S. aureus* mg4n | ST5, CC5 | Gift C. Wolz |
| *S. aureus* mg11c | ST5, CC5 | Gift C. Wolz |
| *S. aureus* USA300 JE2 | ST8, CC8, parental Strain Nebraska mutant library | (Fey et al., 2013) |
| *S. aureus* USA300 JE2 Δ*hsdR* | *hsdR* is deactivated by *bursa aurealis* transposon, Nebraska mutant library | (Fey et al., 2013) |
| *S. aureus* MRSA816 | ST225, CC5 | Gift C. Wolz |
| *S. aureus* Oxford 125 | ST8, CC8 | Peschel strain collection |
| *S. aureus* IVK40 | ST30, CC30, nasal isolate | (Winstel et al., 2015) |
| *S. aureus* IVK90 | ST45, CC45, nasal isolate | (Winstel et al., 2015) |
| *S. aureus* 70153 WT | ΦSebago_int-*tarP* ,ST398 | Jesper Larsen, SSI |
| *S. aureus* 70153 ΦSebago_int-*tarP*::erm | *ermB*-labelled *tarP phage*, ST398 | This study |
| *S. aureus* R5 | Phage indicator strain | (van Wamel et al., 2006) |

**Supplementary Table 4**: Oligonucleotides used in this study

| **Olignucleotide** | **Sequence (5’ -> 3’)** | **Description** |
| --- | --- | --- |
| CC8_hsdR_Bam | ATCGGATCCAAAGGAGGTTATATAATGGCATACCAAAGTGAATACG | Cloning of *hsdR* |
| CC8_hsdR_Sac | GTCGAGCTCTTACACACCGTATTTTTCAGTTGTTT | Cloning of *hsdR* |
| IEC::tet_A | GACGAATTCGTGAAAAGGGTTGTTTATGGGGC | Insertion of tet cassette into IEC of φN315 |
| IEC::tet_B | CTTATATTTTGTTCTAGgATccCTGTGAATAGTCATAGGCGTCCATACATAATC | Insertion of tet cassette into IEC of φN315 |
| IEC::tet_C | GAGTTTTTAGAACAAGgATcCGGTAAAGAAAGTGTTAGGTTACTAGGCCACTTAAC | Insertion of tet cassette into IEC of φN315 |
| IEC::tet_D | ctcGAGCTCCCCTGGATTCAACTTAATTACAAAGG | Insertion of tet cassette into IEC of φN315 |
| Erm_Fw | TACCGTTCGTATAGCATACATTATAC | Insertion of erm-cassette into ΦSa1int-*tarP* |
| Erm_Rv | TTAACCCTAAAGTTATGGAAATAAGAC | Insertion of erm-cassette into ΦSa1int-*tarP* |
| Sa1int_erm_A_2 | gacGGTACCCCCTGGATTCAACTTAATTACAAAG | Insertion of erm-cassette into ΦSa1int-*tarP* |
| Sa1int_erm_B_2 | GTATAATGTATGCTATACGAACGGTATTAACAGGCTATATAGTTCACTCC | Insertion of erm-cassette into ΦSa1int-*tarP* |
| Sa1int_erm_B_3 | CACATTTATAGGATCCTTAACAGGCTATATAGTTCACTCC | Insertion of erm-cassette into ΦSa1int-*tarP* |
| Sa1int_erm_C_3 | GCCTGTTAAGGATCCTATAAATGTGAAATGGTCATTCTTGAAATG | Insertion of erm-cassette into ΦSa1int-*tarP* |
| Erm_BamHI_Fw_2 | CTCGGATCCTACCGTTCGTATAGCATACATTATAC | Insertion of erm-cassette into ΦSebago_int-*tarP* |
| Erm_BamHI_Rv_2 | CCTGGATCCTTAACCCTAAAGTTATGGAAATAAGAC | Insertion of erm-cassette into ΦSebago_int-*tarP* |
| Phi154-3 Ery A | gacGAATTCgagaaggaaaaggaaaataaatcg | Insertion of erm-cassette into ΦSebago_int-*tarP* |
| Phi154-3 Ery B | CTATACGAACGGTAggatccttgattaatttagtgtgtactataaattttac | Insertion of erm-cassette into ΦSebago_int-*tarP* |
| Ery-pBASE_FW | ggatccTAC CGT TCG TAT AGC ATA CAT TAT AC | Insertion of erm-cassette into ΦSebago_int-*tarP* |
| Ery-pBASE_RV | ggtaccTTAACCCTAAAGTTATGGAAATAAGAC | Insertion of erm-cassette into ΦSebago_int-*tarP* |
| Phi154-3 Ery C | CATAACTTTAGGGTTAAggtacctaaacttttcttttgtaacaaacatatttttg | Insertion of erm-cassette into ΦSebago_int-*tarP* |
| Phi154-3 Ery D | ctcGAGCTCagagaataacaaatatcgccttaaca | Insertion of erm-cassette into ΦSebago_int-*tarP* |

## Appendix:

### Phylogeny of CC1, CC45 and ST5 genomes presented in this study with indicated marker genes *mecA*, *mecC*, IEC, *tarM*, *tarP*, *tarS*, and Sa3int

The respective content (*S. aureus* lineage) of the phylogenetic trees files is as follows: Data sheet 1 – CC1; Data sheet 2 – CC45; Data sheet 3 – ST5

### Structural analysis of WTA from *tarM/tarP-*containing *S. aureus* strains (RN4220 ΦN315 and NCTC13132)

Comparison of the proton (Fig. 4B) and heteronuclear spectra (Supplementary Figure 3) of the two strains, RN4220 ΦN135 and NCTC13132, disclosed a strict similarity between their WTAs, therefore, the discussion of the NMR discussion will detail those from RN4220 ΦN135 and will point to the main differences with those of NCTC13132.

Inspection of the proton spectrum of RN4220 ΦN135 (Fig. 4B) showed several signals with no stoichiometric ratio in the region at 5.7 – 4.5 ppm, that normally is diagnostic of the anomeric signals of carbohydrates, a crowded carbinolic region (4.4 – 3.4 ppm), a group of N-acetyl signals at about 2.1 ppm, and the methyl groups of alanine (Ala) residues at about 1.6 ppm.

The identity of all the residues composing the WTA sample was inferred by analysing the complete set of NMR spectra.

First, the analysis of the HSQC spectrum of the region at 5.7 – 4.5 ppm (Supplementary Figure 3a,b) displayed several densities, with only few compatible with the anomeric carbons of the monosaccharide units. These densities were labelled with small letters and based on their ^1^H and ^13^C chemical shift, the units **a** – **c** (Supplementary Table 1) belonged to β-configured units, while **e** – **f** had the α configuration. Then, the HSQC-TOCSY (100 ms, Supplementary Figure 3d) and the TOCSY spectra displayed all the connections from each anomeric proton to the densities of all the other carbons of the unit, a pattern diagnostic of a *gluco* configured residue. Then, the HSQC-TOCSY spectrum (20 ms of mixing time) related each anomeric proton to its carbon 2 (C-2) which was at ca. 57 ppm for the β units and at ca. 55 ppm for the α units. Identification of the other proton and carbon chemical shifts was possible by combining the information from all the NMR spectra along with the literature data (Gerlach, Nature 2018, Xia et al 2010). Overall, this information indicated that all the monosaccharides were terminal N-acetyl-glucosamine units (GlcNAc), either α or β configured at the anomeric centre and as expected.

With regard to the other signals in the anomeric region, the proton and carbon chemical shift values (Supplementary Fig. 3d,e and Supplementary Table 1) were diagnostic of protons shifted at low field due to acylation. Then, each signal was labelled with a capital letter, and the analysis of the HSQC-TOCSY spectrum (100 ms of mixing time) showed that the proton at 5.37 (unit **B**) had only one correlation with a CH_2_-type carbon at 64.9 ppm, a pattern diagnostic of a 1,3-di-phospoglycerol unit acylated at position 2. Conversely, all the other signals correlated with more than one carbon atom, as occurs for the ribitol (Rbo) units. The identification of the **A** – **K** units, which accounted for most of the glycerol (Gro) and Rbo residues of the WTA samples, was possible by comparing the NMR chemical shift with the published data (Gerlach, Nature 2018). Accordingly, the signal at 5.42 ppm was attributed to H-2 of a Rbo (unit **A**) with an alanine (Ala) linked to the corresponding hydroxyl function; the signal at 5.37 ppm was assigned to H-2 of a Gro (**A**) with an Ala esterified at the same position; the signal at 5.24 ppm to H-3 of a Rbo (**C**) with an Ala at O-3. The units **D**, **E** and **F** were identified as di-phosphoribitol, a monophosphoribitol and a diphosphoglycerol units, respectively, based on literature data (Gerlach 2018). The two Rbo units **G** and **H** had an Ala esterified at position two as **A**, but they were glycosylated to O-3 with a β-GlcNAc residue, as inferred from the key correlations, **H_3_b_1_** and **G_3_c_1_** (Supplementary Figure 3e), or **b_1_H_3_** and **c_1_G_3_** (Supplementary Figure 3d), found in the HMBC spectrum. Consistent with the literature data, there were also **I**, **J** and **K**, ascribed to Rbo units with a β-GlcNAc attached to O-3 and differing for having two phosphodiester linkages, or one phosphodiester and one phosphomonoester linkage, or only one phosphodiester linkage, respectively, as depicted in Table cscS1. Finally, the anomeric region contained two other protons shifted because geminal to an ester-linked Ala. The HSQC-TOCSY spectrum recorded with 20 ms of mixing time, showed the correlations of the proton at 5.33 ppm (**L**) with the carbon to which it was directly attached, at 75.8 ppm, and with the two carbon atoms next to it: a CH_2_ at 64.4 and CH at 69.5 ppm. Based on this finding, the proton at 5.33 ppm was identified as H-2 of a Rbo unit (**L**), while the other two densities were assigned to C-1 and C-3 of this polyol. Then, the HSQC-TOCSY acquired with 100 ms, showed two additional densities at 79.0 and 65.5 ppm, that were assigned to C-4 and C-5. The identification of the proton chemical shifts of this Rbo unit was inferred by crossing the information of the HSQC-TOCSY (100 ms) with those of all the other NMR spectra. Finally, the HMBC spectrum had key correlations that interconnected H-1 of α-GlcNAc **d** with C-4 of **L** (marked as **d_1_L_4_** in Supplementary Figure 3e), or H-4 of **L** with C-1 of **d** (**L_4_d_1_** in Supplementary Figure 3c). Thus, **L** was a Rbo unit with an Ala at O-2 and an α-GlcNAc at O-4. By the same approach, it was found that the proton at 5.31 ppm belonged to a Rbo unit (**M**) with an Ala at O-3 and an α-GlcNAc (**e**) at O-4. Finally, the carbinolic area of the HSQC spectrum (Supplementary Figure 3f) contained one density at ^1^H/^13^C 4.08/78.9 ppm, with the carbon value denoting a low field displacement due to glycosylation. This unit was labelled **N** and it was a Rbo glycosylated by α-GlcNAc (**f**) as proved by the correlation **N_4_f_1_** (Supplementary Figure 3c). The attribution of this unit as a Rbo with a α-GlcNAc at O-4 along with the identification of all the other chemical shifts, was possible by matching the densities left unassigned in the spectrum with those from literature (Xia 2010).

Thus, the NMR study identified the major motifs occurring in each WTA sample and that are reported in Supplementary Table 1, while it was not possible to define the nature of minor motifs since their signals were weak and their correlations merged into those of the more abundant motifs.

With regard to the WTA from the strain NCTC13132, its NMR spectra matched those recorded for RN4220 ΦN135 and the only differences regarded the relative ratio between the different motifs. In order to pinpoint such differences, the proportions between the different motifs were calculated by integrating the key densities of the HSQC spectrum of each sample, according to the approach developed previously (Gerlach 2018). As result, the two samples showed some subtle differences (Supplementary Table 2): the ratio Rbo/Gro was slightly higher in NCTC13132, whose WTA was more glycosylated and with a higher proportion of β-GlcNAc compared to the WTA of NCTC13132.

**Supplementary References**

Brown, S., Xia, G., Luhachack, L.G., Campbell, J., Meredith, T.C., Chen, C., et al. (2012). Methicillin resistance in Staphylococcus aureus requires glycosylated wall teichoic acids. *Proc Natl Acad Sci U S A* 109(46)**,** 18909-18914. doi: 10.1073/pnas.1209126109.

Enright, M.C., Day, N.P., Davies, C.E., Peacock, S.J., and Spratt, B.G. (2000). Multilocus sequence typing for characterization of methicillin-resistant and methicillin-susceptible clones of Staphylococcus aureus. *J Clin Microbiol* 38(3)**,** 1008-1015. doi: 10.1128/JCM.38.3.1008-1015.2000.

Fey, P.D., Endres, J.L., Yajjala, V.K., Widhelm, T.J., Boissy, R.J., Bose, J.L., et al. (2013). A genetic resource for rapid and comprehensive phenotype screening of nonessential Staphylococcus aureus genes. *mBio* 4(1)**,** e00537-00512. doi: 10.1128/mBio.00537-12.

Gerlach, D., Guo, Y., De Castro, C., Kim, S.H., Schlatterer, K., Xu, F.F., et al. (2018). Methicillin-resistant Staphylococcus aureus alters cell wall glycosylation to evade immunity. *Nature* 563(7733)**,** 705-709. doi: 10.1038/s41586-018-0730-x.

Kreiswirth, B.N., Lofdahl, S., Betley, M.J., O'Reilly, M., Schlievert, P.M., Bergdoll, M.S., et al. (1983). The toxic shock syndrome exotoxin structural gene is not detectably transmitted by a prophage. *Nature* 305(5936)**,** 709-712. doi: 10.1038/305709a0.

Kuroda, M., Ohta, T., Uchiyama, I., Baba, T., Yuzawa, H., Kobayashi, I., et al. (2001). Whole genome sequencing of meticillin-resistant Staphylococcus aureus. *The Lancet* 357(9264)**,** 1225-1240. doi: 10.1016/s0140-6736(00)04403-2.

van Wamel, W.J., Rooijakkers, S.H., Ruyken, M., van Kessel, K.P., and van Strijp, J.A. (2006). The innate immune modulators staphylococcal complement inhibitor and chemotaxis inhibitory protein of Staphylococcus aureus are located on beta-hemolysin-converting bacteriophages. *J Bacteriol* 188(4)**,** 1310-1315. doi: 10.1128/JB.188.4.1310-1315.2006.

Winstel, V., Kuhner, P., Salomon, F., Larsen, J., Skov, R., Hoffmann, W., et al. (2015). Wall Teichoic Acid Glycosylation Governs Staphylococcus aureus Nasal Colonization. *MBio* 6(4)**,** e00632. doi: 10.1128/mBio.00632-15.

Xia, G., Corrigan, R.M., Winstel, V., Goerke, C., Grundling, A., and Peschel, A. (2011). Wall teichoic Acid-dependent adsorption of staphylococcal siphovirus and myovirus. *J Bacteriol* 193(15)**,** 4006-4009. doi: 10.1128/JB.01412-10.
